# Pterostilbene in Combination With Mitochondrial Cofactors Improve Mitochondrial Function in Cellular Models of Mitochondrial Diseases

**DOI:** 10.3389/fphar.2022.862085

**Published:** 2022-03-18

**Authors:** Juan M. Suárez-Rivero, Carmen J. Pastor-Maldonado, Ana Romero-González, David Gómez-Fernandez, Suleva Povea-Cabello, Mónica Álvarez-Córdoba, Irene Villalón-García, Marta Talaverón-Rey, Alejandra Suárez-Carrillo, Manuel Munuera-Cabeza, José A. Sánchez-Alcázar

**Affiliations:** Centro Andaluz de Biología Del Desarrollo (CABD-CSIC-Universidad Pablo de Olavide), Centro de Investigación Biomédica en Red: Enfermedades Raras, Instituto de Salud Carlos III, Sevilla, Spain

**Keywords:** pterostilbene, sirt3, UPR^mt^, mitochondrial diseases, mitochondrial cofactors

## Abstract

Mitochondrial diseases are genetic disorders caused by mutations in genes in the nuclear DNA (nDNA) and mitochondrial DNA (mtDNA) that encode mitochondrial structural or functional proteins. Although considered “rare” due to their low incidence, such diseases affect thousands of patients’ lives worldwide. Despite intensive research efforts, most mitochondrial diseases are still incurable. Recent studies have proposed the modulation of cellular compensatory pathways such as mitophagy, AMP-activated protein kinase (AMPK) activation or the mitochondrial unfolded protein response (UPR^mt^) as novel therapeutic approaches for the treatment of these pathologies. UPR^mt^ is an intracellular compensatory pathway that signals mitochondrial stress to the nucleus for the activation of mitochondrial proteostasis mechanisms including chaperones, proteases and antioxidants. In this work a potentially beneficial molecule, pterostilbene (a resveratrol analogue), was identified as mitochondrial booster in drug screenings. The positive effects of pterostilbene were significantly increased in combination with a mitochondrial cocktail (CoC3) consisting of: pterostilbene, nicotinamide, riboflavin, thiamine, biotin, lipoic acid and l-carnitine. CoC3 increases sirtuins’ activity and UPR^mt^ activation, thus improving pathological alterations in mutant fibroblasts and induced neurons.

## Introduction

Mitochondrial diseases encompass a broad spectrum of muscular and neurodegenerative disorders, chronic and progressive, caused by mutations in nuclear (nDNA) or mitochondrial (mtDNA) DNA (Zeviani and Carelli, 2007). The prevalence of these diseases has been established at 1:5,000 (Gorman et al., 2015). Most oxidative phosphorylation disorders in children area consequence of the mutation of nuclear DNA, which are transmitted as autosomal recessive traits, usually with severe phenotypes and a fatal outcome. Among the maternally-inherited pathogenic mtDNA mutations, more than 50% have been identified in genes encoded by mitochondrial transfer RNAs (mt-tRNA) (MTT genes).

To maintain protein homeostasis (proteostasis), mitochondria possess a complex quality control machinery. Proteostasis is defined as a cellular state at which protein synthesis, folding and degradation remain in equilibrium, which is only altered upon condition-specific cellular demands (Sala et al., 2017). These processes are essential for mitochondrial function, given that such organelles rely on the functionality of approximately 1500 targeted proteins (Pfanner et al., 2019). In fact, the inner mitochondrial membrane presents an uncommon protein-lipid ratio of 80:20 (Giacomello et al., 2020). Interestingly, 99% of mitochondrial proteins are nDNA encoded and imported from the cytoplasm, while the mitochondrial genome only encodes 13 proteins (Endo et al., 2011). This implies that most mitochondrial proteins must be imported and assembled into respiratory complexes in order to be biologically active. Such process is highly regulated but not infallible. The accumulation of incomplete respiratory complexes and the increase of intracellular reactive oxygen species (ROS) are, indeed, common features among mitochondrial disease patients (Suhm et al., 2018). Furthermore, it has recently been discovered that the mitochondrial proteome is subject to substantial changes depending on the availability of nutrients, cellular stress or the presence of toxins, as a mechanism to adapt mitochondrial function to different adversities (Pfanner et al., 2019).

The loss of mitochondrial proteostasis has been directly linked to ageing and its associated illnesses (Klaips et al., 2018; Hipp et al., 2019), neurodegeneration (Chiti and Dobson, 2017; Hou et al., 2019), ROS overproduction (Quiles and Gustafsson, 2020) and mitochondrial diseases (Johnson et al., 2013; Sorrentino et al., 2018). Failure in the proteostasis machinery leads to diverse protein alterations such as accumulation of aggregates (Sorrentino et al., 2017) or premature degradation (Ruan et al., 2020), thus triggering mitochondrial dysfunction. In order to palliate these defects, mitochondria activate compensatory mechanisms such as mitophagy or the mitochondrial unfolded protein response (UPR^mt^). Mitophagy is defined as a selective autophagy process targeting damaged mitochondria, and it has proven to be of essential importance in the renewal of these compartments and thus, the maintenance of cellular fitness (Ashrafi and Schwarz, 2013; Yoo and Jung, 2018). Functional mitophagy is indispensable for the degradation of aberrant mitochondria, reason why boosting such mechanism has been proposed as a strategy to exert selective pressure over mutant mitochondria on heteroplasmic mitochondrial diseases, at which mutant and wild type mtDNA coexist within a cell (Diot et al., 2015; Villanueva Paz et al., 2016). Up to the present, these assays have only been carried out *in vitro.*

There are currently no effective treatments available for mitochondrial diseases, irrespective of whether they are provoked by mutations on nuclear or mtDNA (Hirano et al., 2018). Given that gene therapies are still far from the clinic (Russell et al., 2020), the therapeutic approaches for this set of diseases are evolving from the sustained administration of mitochondrial cofactors cocktails to treatments with activators of mitochondrial compensatory mechanisms, such as the UPR^mt^. Several studies support that this latter is in fact a promising approach, both *in vitro* and *in vivo* (Houtkooper et al., 2013; Jenkins et al., 2021; Perry et al., 2021). Notwithstanding, such studies suggest that treatment with antibiotics as a strategy to activate mitochondrial compensatory pathways. However, the chronic administration of antibiotics to patients is highly controversial, the debate being whether their side effects might outweigh their beneficial properties (Suarez-Rivero et al., 2021). In this context, our aim was to identify non-antibiotic FDA-approved alternative drugs to boost UPR^mt^ activation in patients’ cells so that the defects derived from mitochondria-associated mutations are compensated. Pterostilbene is a natural phytoestrogen, a dimethyl ether resveratrol analogue, with a higher bioavailability that is able to cross the blood-brain barrier (Wang and Sang, 2018). This polyphenol is considered to be safe for human consumption and presents numerous well-known features such as a prominent antioxidant activity and a high anti-inflammatory potential (Lange and Li, 2018). Furthermore, it has been reported to prolong lifespan in several animal models (Li YR. et al., 2018) due to its neuroprotective (Li Q. et al., 2018) and cardioprotective (Natalin et al., 2016) properties. At molecular level pterostilbene activates sirtuins (Li YR. et al., 2018) and has additionally been linked to AMPK (Malik et al., 2019) and Nrf2 by recent studies (Zhou et al., 2019), which therefore suggest that it is a potential compound for maintaining mitochondrial homeostasis.

The goal of this study is to investigate the potential use of pterostilbene as a treatment for mitochondrial diseases. In this line, a series of assays were performed on six different mitochondrial diseases patient-derived fibroblasts in an aim to assess whether the application of such drugs has a beneficial effect on cellular homeostasis and if so, to find out their molecular mechanisms of action.

## Material and Methods

### Reagents

The following antibodies were purchased from Abcam (Cambridge, United Kingdom): mt-CO2 (ab79393), VDAC (ab14734), actin (ab8226), COX4 (ab14744), ATF5 (ab184923), SIRT3 (ab217319), COX15 (ab201082), NDUFS4 (ab137064), NAMPT (ab236874), ATF4 (ab184909), CHOP (ab11419), Nrf2 (ab62352), NDUFA9 (ab14713), SIRT1 (ab110304). COQ7 antibody (NBP1-98496) was purchased from Novus Biologicals (Colorado, CO, United States). NDUFAF6 antibody (DPABH-03128) was purchased from Creative Diagnostics (New York, United States). mtND1 (MBS9402205) and mtND3 (MBS8551680) antibodies were purchased from MyBioSource (San Diego, CA, United States). mtHSP70 antibody (MA3-028), HSP60 antibody (MA3-012), Tau antibody (MN1000), MitoTracker Deep Red FM (M22426), Donkey anti-Rabbit IgG (H + L) Highly Cross-Adsorbed Secondary Antibody, Alexa Fluor 555 (A-31572) and Donkey anti-Mouse IgG (H + L) Highly Cross-Adsorbed Secondary Antibody, Alexa Fluor 488 (A-21202) were purchased from Thermo Fisher (Waltham, MA, United States). eif2α (5324), P-eif2α (9721), mtND3 (82,933), anti-acetylated lysine 9441) antibodies were purchase from Cell Signaling (Danvers, MA, United States). Galactose (sc-202564), paraformaldehyde (sc-253236B), oligomycin (sc-203342), antimycin A (sc-202467A), FCCP (sc-203578), DAPI (sc-3598), nicotinamide (sc-208096), riboflavin (sc-205906), thiamine (sc-205859), L-carnitine (sc-205727) and HEPES (sc-29097) were purchased from Santa Cruz Biotechnology (Santa Cruz, CA, United States). Saponin (S7900-25G), valproic acid (P4543-10G), LDN-1931189 (SML0559-5MG), Db-cAMP (D0260-100MG), CHIR99021 (SML1046-5MG), Goat Anti-Rabbit IgG H&L (HRP) (401353-2ml), Goat Anti-Mouse IgG, H&L Chain Specific peroxidase Conjugate (401253-2ml) and donkey serum (D9663) were purchased from Merck (Darmstadt, Germany). Pterostilbene and 3-TYP was purchased from Cayman Chemical (Ann Harbor, MI, United States). SB431542 (1614/10), Noggin (6057-NG-100), LM-22A4 (4607/10), GDNF (212-GD-010) and NT3 (267-N3-025) were purchased from R&D systems (Minneapolis, MN, United States). PBS (Phosphate Buffer Saline, 102309) 10x was purchased from Intron Biotechnology (Seongnam, South Korea) and then diluted to 1× PBS pH 7.4. BSA (Bovine Serum Albumin, A6588.0100) was purchased from Applichem (Darmstadt, Germany).

### Fibroblast Cultures

Cultured fibroblasts were derived from a skin biopsy of patients (COQ7, COX15, NDUFAF6, NDUFS1, ND3 and NDUFS4) with the following mutations: COQ7: Compound heterozygous mutation in *COQ7* gene; c.161_161deIG (p.Val55fs) and c.319C>T (p.Arg107Trp). COX15: Homozygous mutation in *COX15* gene; c.C649T (p.Arg217Trp). NDUFAF6: Compound heterozygous mutation in *NDUFAF6* gene; c.371T>C (p.Ile124Thr) and c.554_558delTTCTT (p.Tyr187AsnfsTer65). NDUFS1: Homozygous mutation in *NDUFS1* gene; c.A755G (p.Asp252Gly). ND3: Mitochondrial heteroplasmic mutation in *MT-ND3* gene with ∼80% heteroplasmy; m.10191T>C (p.Ser45Pro). NDUFS4: Homozygous mutation in *NDUFS4* gene; c.350+5G>A.

Control fibroblasts were human skin primary fibroblasts from healthy volunteer donors. Samples from patients and controls were obtained according to the Helsinki Declarations of 1964, as revised in 2001. Fibroblasts from patients and controls were cultured at 37°C in DMEM (Dulbecco’s Modified Eagle Medium) containing 4.5 gL^−1^ glucose, L-glutamine, and pyruvate supplemented with 1% antibiotic Pen-Strep solution (Thermo Fisher, Waltham, MA, United States) and 20% Fetal Bovine Serum (FBS) (Thermo Fisher, Waltham, MA, United States). All the experiments were performed with fibroblasts on a passage number lower than 8.

### Drug Screening

Drug screening was performed in restrictive culture medium with galactose as main carbon source. Our aim was to deprive cells from glycolysis as energy source (due to the use of galactose) and hence have them rely exclusively on the mitochondrial electron transport chain for ATP production (Coelho et al., 2015; Kamalian et al., 2019). In this condition, their inherent mutations would make mitochondrial patients’ fibroblasts unable to survive in such medium.

Galactose medium was prepared with DMEM without glucose and glutamine and supplemented with 10 mM galactose, 1% antibiotic solution and 10% FBS. Cells were seeded in 24-well plates in DMEM glucose. After 24 h cells were treated for 72 h with several compounds. Then the medium was removed, and cells were washed with PBS prior to the addition of the galactose medium(T0). Then, the treatments were re-applied in the same concentration.

Cell viability was assessed by live cell imaging counting and trypan blue 0.2% staining. Cell counting and representative images were acquired using the BioTek™ Cytation™ 1 Cell Imaging Multi-Mode Reader (Biotek, Winooski, VT, United States).

### Immunoblotting

Western blotting was performed using standard methods. After transferring the proteins to nitrocellulose membranes (BIORAD, Hercules, CA, United States, #1620115), these were incubated with primary antibodies, which were diluted 1:1,000 in BSA 5% overnight. Then washed twice with TTBS and incubated with the corresponding secondary antibody for 1h at 4°C. Secondary antibodies were diluted 1:2,500 in BSA 5%. Multiple blots were run and several proteins of interest were serially detected. Every membrane was checked for protein loading using Ponceau staining and actin protein levels. Stripping was not used. If possible, membranes were re-probed with different antibodies. This is when the molecular weight of the new protein of interest did not interfere with that of the previous one. Moreover, if the proteins were sufficiently separated from one another during gel electrophoresis, membranes were cut and each respective piece was used to detect a different target protein.

### Bioenergetics

Mitochondrial respiratory function of control and mutant fibroblasts was measured using a mito-stress test assay with an XF24 extracellular flux analyzer (Seahorse Bioscience, Billerica, MA, United States, 102340-100) according to the manufacturer’s instructions. Cells were seeded at a density of 1.5 × 10^4^ cells/well with 500 µl growth medium (DMEM medium containing 20% FBS) in XF24 cell culture plates and incubated for 24 h at 37°C, 5% CO_2_. Subsequently, growth medium was removed from the wells, leaving on them only 50 µl medium. Then, cells were washed twice with 500 µl of pre-warmed assay XF base medium (102353-100) supplemented with 10 mM glucose (103577-100), 1 mM glutamine (103579-100) and 1 mM sodium pyruvate (103578-100); pH 7.4) and eventually 450 µl of assay medium (500 µl final) were added. Cells were incubated at 37°C without CO_2_ for 1 h to allow pre-equilibrating with the assay medium. Mitochondrial functionality was evaluated by sequential injection of four compounds affecting bioenergetics. The final concentrations of the injected reagents were: 1 µM oligomycin, 2 µM FCCP, 1 and 2.5 µM rotenone/antimycin A. The best concentration of each inhibitor and uncoupler, as well as the optimal cells seeding density were determined in preliminary analyses. A minimum of five wells per treatment were used in any given experiment. This assay allowed for an estimation of basal respiration, maximal respiration and spare respiratory capacity. The studied parameters were the following: 1) Basal respiration: Oxygen consumption rate (OCR) used to meet cellular ATP demand resulting from mitochondrial proton leak. It shows energetic demand of the cell under baseline conditions. 2) ATP Production: This parameter is calculated by subtracting the OCR after oligomycin injection from the basal respiration. It shows ATP produced by the mitochondria that contributes to meeting the energetic needs of the cell. 3) Maximal respiration: The maximal OCR attained by adding the uncoupler FCCP. FCCP mimics a physiological “energy demand” by stimulating the respiratory chain to operate at maximum capacity to meet this metabolic challenge. It shows the maximum rate of respiration that the cell can achieve. 4) Spare respiratory capacity: This measurement indicates the capability of the cell to respond to an energetic demand as well as how closely the cell is to respire to its theoretical maximum.

### Mitochondrial Complexes Activity

Activity of complex I and complex IV was assessed according to the protocol of the Complex I (ab109720)/Complex IV (ab109876) Enzyme Activity Dipstick Assay Kit (Abcam, Cambridge, United Kingdom). In this technique the proteins from cellular lysates migrate through a nitrocellulose membrane. Then, Complex I is immunocaptured (i.e., immuno-precipitated in active form) on the dipstick. Then, the dipstick is immersed in Complex I activity buffer solution containing NADH as a substrate and nitrotetrazolium blue (NBT) as the electron acceptor. Immunocaptured complex I oxidizes NADH and the resulting H+ reduces NBT to form a blue-purple precipitate at the Complex I antibody line on the dipstick. The signal intensity of this precipitate corresponds to the level of Complex I enzyme activity in the sample. Dipsticks images were taken with a Molecular Imager ChemiDoc XRS+ System (BIORAD, Hercules, CA, United States) and quantified by the Image Lab software.

### NAD^+^/NADH Levels

NAD^+^/NADH levels in cellular pellets were assessed by the NAD^+^/NADH Colorimetric Assay Kit (Abcam, Hercules, CA, United States, ab65348) protocol. Colour intensity was measured using a POLARstar Omega plate reader (BMG Labtech, Offenburg, Germany).

### SIRT3 Activity

Mitochondrial extracts were obtained using the Mitochondrial Isolation Kit for Cultured Cells (Abcam, Hercules, CA, United States, ab110170). Then, SIRT3 activity was determined by the SIRT3 Fluorometric Activity Assay Kit (Abcam, Hercules, CA, United States, ab156067) protocol. Fluorescence was measured using a POLARstar Omega plate reader (BMG Labtech, Offenburg, Germany).

### Direct Reprogramming

Neurons were generated from mutant NDUFAF6 and control fibroblasts by direct neuronal reprogramming as previously described by Drouin-Ouellet et al. [28, 29]. Controls and mutant NDUFAF6 patient-derived fibroblasts were plated on μ-Slide 4-Well Ibidi plates (Ibidi, Gräfelfing, Germany) and cultured in DMEM + Glutamax (61965059) with 1% Pen-Strep solution and 10% FBS.

The day after, dermal fibroblasts were transduced with one-single lentiviral vector containing neural lineage-specific transcription factors (ASCL1 and BRN2) and two shRNA against the REST complex, which were generated as previously described with a non-regulated ubiquitous phosphoglycerate kinase (PGK) promoter [30]. The plasmid was a gift from Dr. Malin Parmar (Developmental and Regenerative Neurobiology, Lund University, Sweden). Transduction was performed at a multiplicity of infection (MOI) of 30. On the following day cell culture medium was switched to fresh DMEM and after 48 h to neural differentiation medium (NDiff227; Takara-Clontech, Kusatsu, Japan, Y40002) supplemented with neural growth factors and small molecules at the following concentrations: LM-22A4 (2 μM), GDNF (2 ng/ml), NT3 (10 ng/ml), dibutyryl cyclic AMP (db-cAMP, 0.5 mM), CHIR99021 (2 μM), SB-431542 (10 μM), noggin (50 ng/ml), LDN-193189 (0.5 M) and valproic acid (VPA, 1 mM). Half of the neuronal differentiation medium was refreshed every 2–3 days. Eighteen days post-infection (DPI), the medium was replaced by neuronal medium supplemented with only growth factors until the end of the cellular conversion. At day 21, cells were treated with CoC3 and the medium was changed every 2–3 days for 10 more days. Neuronal cells were identified by the expression of Tau protein, using the anti-TAU clone HT7 antibody from Invitrogen/Molecular Probes (Eugene, OR, United States). Nuclei were stained with DAPI (Invitrogen/Molecular Probes, Eugene, OR, United States, D1306). DAPI+/Tau+ cells were considered iNs. Conversion efficiency was calculated as the number of Tau+ cells over the total number of fibroblasts seeded for conversion. Neuronal purity was calculated as the number of Tau+ cells over the total cells in the plate after reprogramming.

### Immunofluorescence Microscopy

Treated and untreated fibroblasts were grown on 1 mm width glass coverslips for 72 h in normal growth medium with/without the addition of CoC3. Cells were stained with 100 nM MitoTracker DeepRed FM 45 min before fixation. Afterwards, they were washed twice with PBS, fixed in 3.8% paraformaldehyde for 15 min at room temperature, incubated in blocking buffer (BSA 1% in PBS) and permeabilized with 0.1% saponin in blocking buffer for 1 h. In the meantime, primary antibodies were diluted 1:100 in antibody buffer (BSA 0.5% plus saponin 0.1% in PBS). Fibroblasts were incubated overnight at 4°C with the antibodies and subsequently washed twice with PBS. Secondary antibodies were similarly diluted 1:400 antibody buffer, but their incubation time on cells was reduced to 2 h at room temperature. Coverslips were then washed twice with PBS, incubated for 5 min with PBS containing DAPI 1 μg/ml and washed again with PBS. Next, they were mounted on microscope slides using Vectashield Mounting Medium (Vector Laboratories, Burlingame, CA, United States, H1000).

Neurons were grown on 4 μ-SLIDE 4-well plates (Ibidi, Gräfelfing, Germany, 80,426) and stained with 100 nM MitoTracker Deep Red FM 45 min before fixation. Cells were washed with PBS, fixed in 4% paraformaldehyde for 10 min at room temperature, and permeabilized with 0.1% Triton X-100 for 10 min. Then, blocking buffer consisting of PBS 5% donkey serum was added for 1 h. Primary antibodies were diluted 1:100 in PBS 5% donkey serum and incubated on the cells overnight at 4°C. The following morning neurons were washed twice with PBS prior to the addition of the secondary antibodies. These were diluted 1:300 in PBS 5% donkey serum and incubated for 2 h at room temperature. Finally, cells were washed twice with PBS, incubated for 15 min with PBS containing DAPI dilution 1 μg/ml and washed with PBS.

Samples were analyzed using an upright fluorescence microscope (Leica DMRE, Leica Microsystems GmbH, Wetzlar, Germany). Images were taken using a DeltaVision system (Applied Precision; Issaquah, WA, United States) with an Olympus IX-71 microscope using a ×100 objective. Images were analysed using the softWoRx and ImageJ software.

### Statistical Analysis

We used non-parametric statistics, which do not consider distributional assumptions, given the low reliability of normality testing for small sample sizes like those used in this work (Le Boedec, 2016). To compare parameters between groups, variables were evaluated using the Wilcoxon match-paired signed rank test, the Friedman Test or a 2-way ANOVA Test. All results were expressed as mean ± standard deviation (SD) of three independent experiments and a *p*-value < 0.05 was considered as statistically significant.

## Results

### Pterostilbene and Mitochondrial Cofactors Supplementation Enables the Survival of Mitochondrial Mutant Fibroblasts in Galactose Medium

**FIGURE 1:**
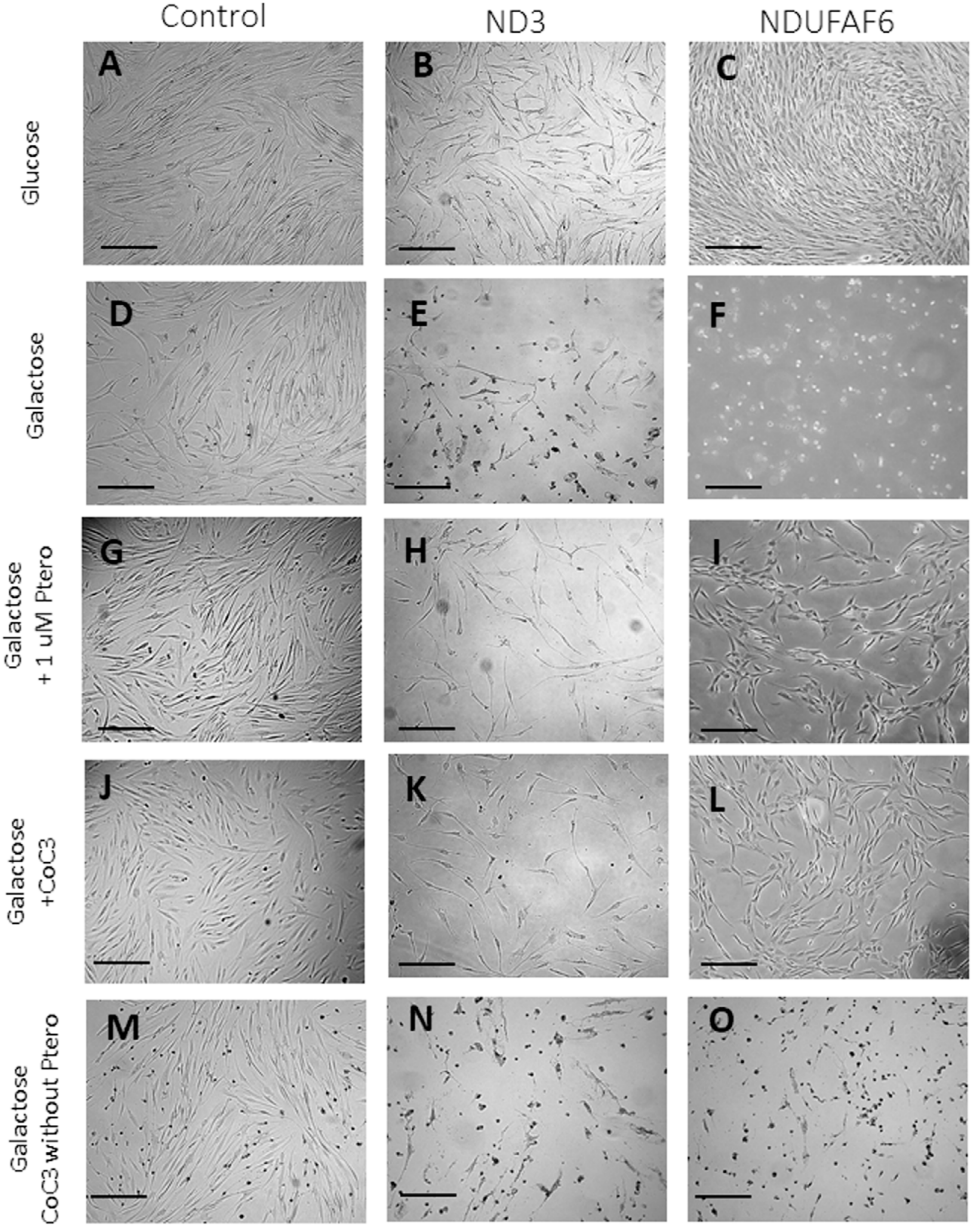
Screening of compounds in galactose medium. Cells were initially seeded in DMEM high glucose. After 3 days, glucose medium was changed to galactose. Images were acquired right after changing the medium and after 72 h of incubation. In optimal conditions control and both mutant cell lines present a similar proliferation rate **(A–C)**. Control cells almost do not alter their growth rate **(D)** but mutant ND3 and NDUFAF6 cells are unable to survive **(E and F)**. Pterostilbene (1 μM) treatment does not affect control cells **(J)** but promotes the survival of mutant ND3 and NDUFAF6 cells in galactose medium **(H and I)**. In addition, cocktail CoC3 (1 μM Pterostilbene, 5 μM nicotinamide, 1 μM riboflavin, 1 μM thiamine, 1 μM biotin, 5 μM lipoic acid and 1 μM L-carnitine) significantly improves pterostilbene’s positive effects **(J–L)**. CoC3 without ptersotilbene was also examined, but no favorable effect could be observed on mutant fibroblast **(N and O)**, suggesting that pterostilbene is necessary for the positive effect of CoC3. The quantification of cellular proliferation is shown in Supplementary Figure S4. Scale bar = 40 μm.

Control cells and mutant mtND3 or NDUFAF6 cells were cultured for 3 days on glucose-rich DMEM medium. Then, the medium was replaced by galactose medium. As expected, almost no differences could be observed on the growth rate of control cells after the switch to galactose (Figures 1A,D). In contrast, mtND3 and NDUFAF6 mutant cells did not survive in galactose medium (Figures 1E,F). Interestingly, 1 μM pterostilbene treatment, a SIRT3 activator (Li YR. et al., 2018), enabled the survival of mtND3 and NDUFAF6 cells on galactose medium, even though their growth rate was slower than before (Figures 1H,I). The efficacy of pterostilbene was enhanced when it was co-administered with mitochondrial cofactors: nicotinamide 5 μM (Canto et al., 2012), riboflavin 1 μM (Henriques et al., 2016), thiamine 1 μM (Depeint et al., 2006), biotin 1 μM (Rodriguez-Fuentes et al., 2007), lipoic acid 5 μM (Solmonson and DeBerardinis, 2018) and L-carnitine 1 μM (Figures 1K,L). This cocktail plus pterostilbene 1 μM was named CoC3. In contrast, treatment with CoC3 without pterostilbene was insufficient to promote mutant fibroblasts survival and/or growth (Figures 1N,O), indicating that pterostilbene is necessary for CoC3 beneficial effects. Also, individual treatment of each CoC3 component was unable to promote cell survival by their own (Supplementary Figures S1, S2).

To assess the role of SIRT3 activation under CoC3 supplementation control and mutant cells were treated with 3-TYP, a SIRT3 inhibitor, in all experimental conditions. No differences were observed in control cells on glucose or galactose medium (Supplementary Figures S3A,D, S4A). However, mutant cells cultured in galactose medium did not survive even with CoC3 supplementation (Supplementary Figures S3E,F,H,I,K,L, S4B,C). This data suggests that SIRT3 activation may play a crucial role in mutant cell survival and the mechanism of action of pterostilbene and CoC3.

### Pterostilbene and Mitochondrial Cofactors Supplementation Restores Protein Expression Pattern in Several Mitochondrial Patient Cell Lines

**FIGURE 2:**
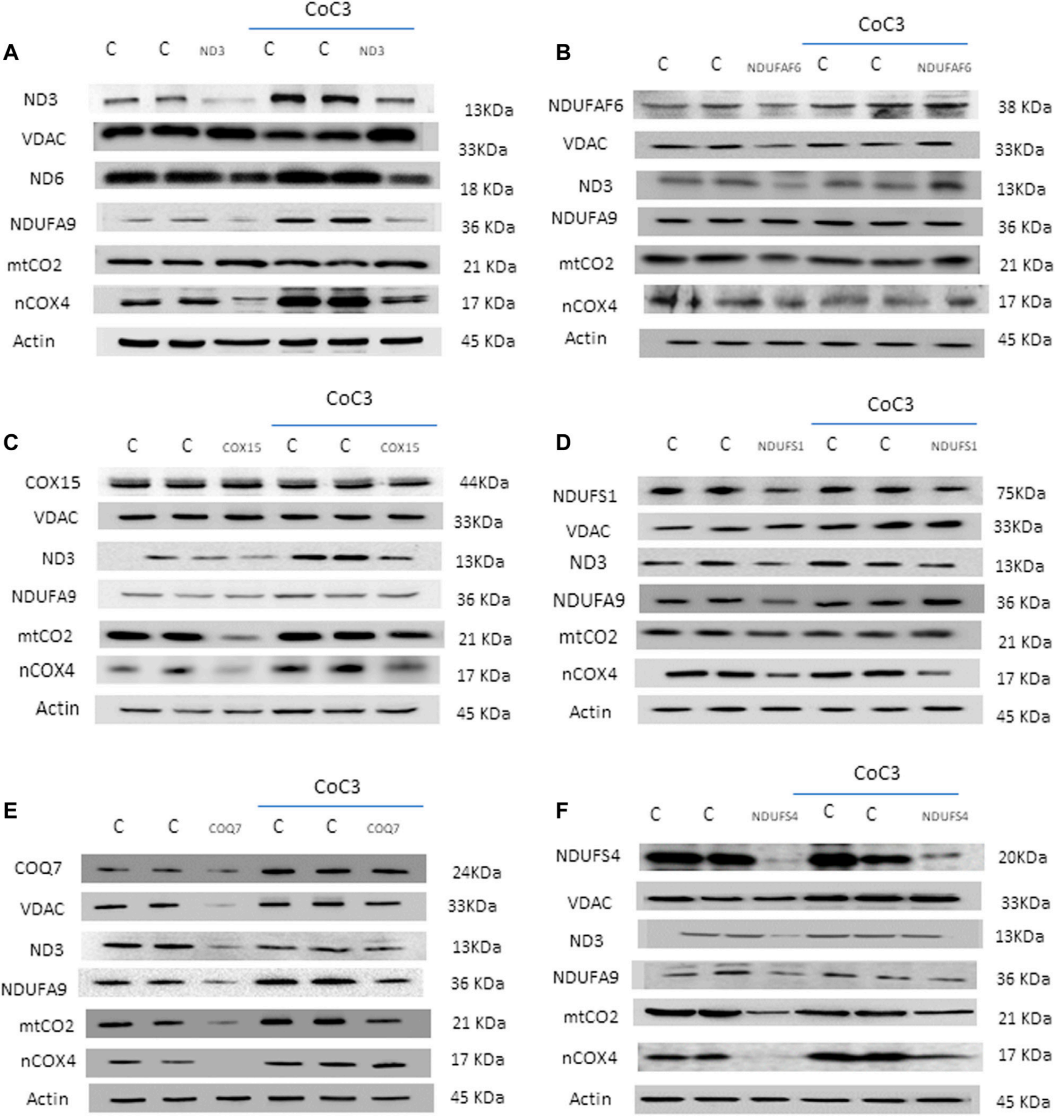
Effect of CoC3 on mitochondrial protein expression levels in control and mutant fibroblasts. Western blot analysis of the affected mutant protein in each cell line: mtND3 **(A)**, NDUFAF6 **(B)**, COX15 **(C)**, NDUFS1 **(D)**, COQ7 **(E)**, NDUFS4 **(F)**. VDAC was used as mitochondrial mass marker, mtND3 and mtCO2 as mitochondrial-encoded reference proteins and NDUFA9 and nCOX4 as nuclear-encoded mitochondrial reference proteins. Overall, all patients show alterations with respect to the control, which are partially reversed after CoC3 treatment. “C” stands for healthy control cells. CoC3 treatment: 1 μM Pterostilbene, 5 μM nicotinamide, 1 μM riboflavin, 1 μM thiamine, 1 μM biotin, 5 μM lipoic acid and 1 μM L-carnitine. Differences between patients are probably due to the high variability in mutation severity and their genetic background. A representative actin band is shown, although loading control was additionally checked for every Western blot. Band densitometry can be found in Supplementary Figure S5.

The positive effect of CoC3 treatment was evaluated in six patient cell lines bearing mutations in mtND3 (Figure 2A), NDUFAF6 (Figure 2B), COX15 (Figure 2C), NDUFS1 (Figure 2D), COQ7 (Figure 2E) and NDUFS4 (Figure 2F).

First, mitochondrial protein expression levels were assessed in the 6 cell lines via Western blotting. For each mutant cell line, the affected protein as well as several critical mitochondrial proteins were examined: subunits of complex I (mtND3 and NDUFA9), subunits of complex IV (mtCO2 and nCOX4), VDAC as mitochondrial mass marker and actin as loading control. In addition, the changes in protein expression levels upon treatment with CoC3 were evaluated. Given the broad diversity in genetic backgrounds and mutations, results varied among the mutant cell lines. Notwithstanding, most mitochondrial proteins presented lower expression levels in all mutant cell lines, though to different extents. Interestingly, treatment with CoC3 significantly enhanced mitochondrial protein expression levels in all mutant cell lines (Figures 2A,F; Supplementary Figures S5A–F).

**FIGURE 3:**
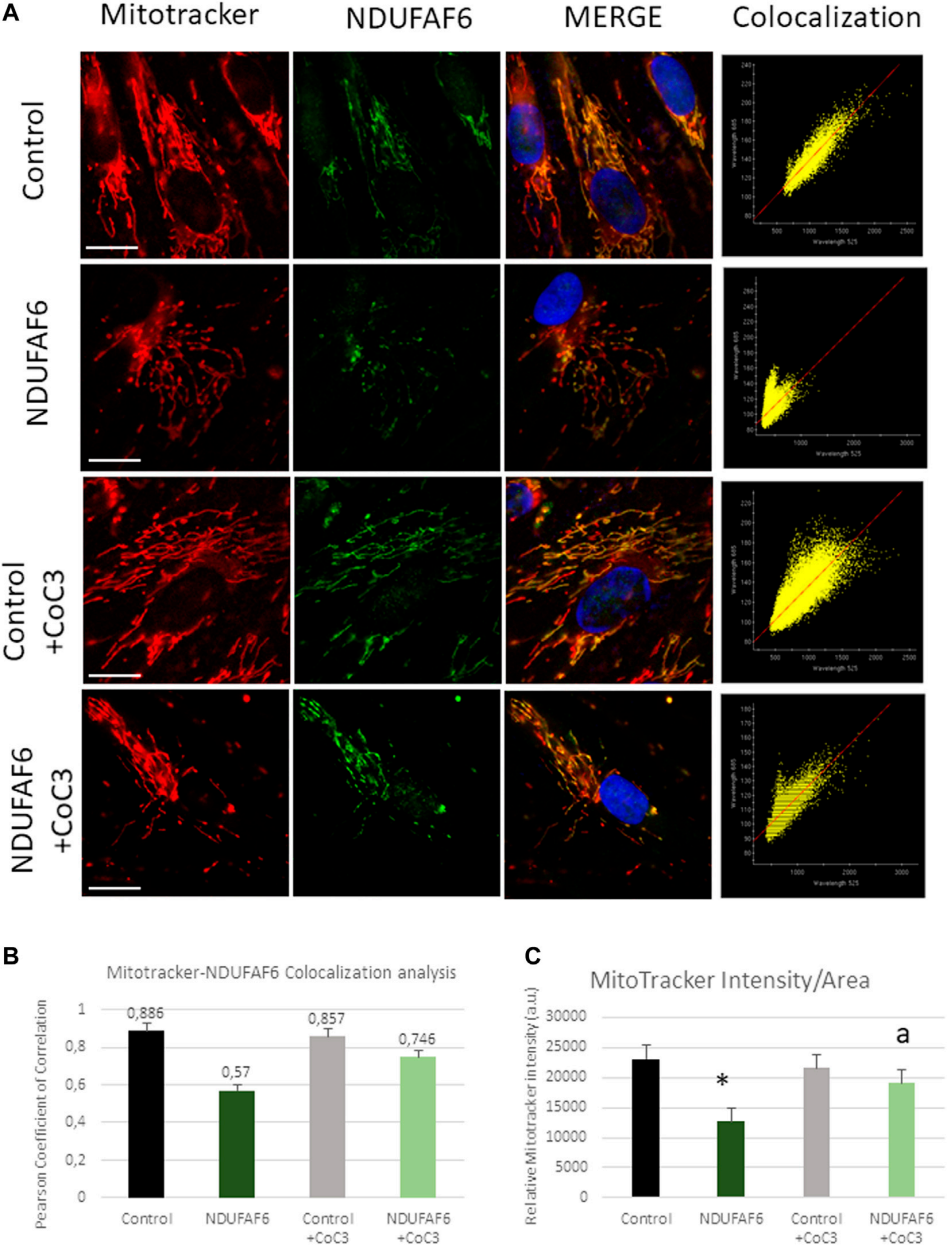
Effect of CoC3 on NDUFAF6 protein expression levels and mitochondrial network in control and mutant NDUFAF6 fibroblasts. Control and mutant NDUFAF6 fibroblasts were incubated with Mitotracker DeepRed FM 100 nM for 45 min, then they were fixed and immunostained with anti-NDUFAF6 (NADH:Ubiquinone oxidoreductase Complex Assembly Factor 6) and examined by fluorescence microscopy. Nuclei were revealed by 1 μg/ml Hoechst staining **(A)**. Colocalization analyses **(B)** and MitoTracker **(C)** intensity assessment were performed using softWoRx and ImageJ softwares. CoC3 treatment: 1 μM Pterostilbene, 5 μM nicotinamide, 1 μM riboflavin, 1 μM thiamine, 1 μM biotin, 5 μM lipoic acid and 1 μM l-carnitine. *p < 0.05 between control and mutant NDUFAF6 cells; ^a^ *p* < 0.05 between non-treated mutant NDUFAF6 and treated mutant NDUFAF6 cells. Scale bar = 15 μm.

The efficacy of CoC3 treatment in the mutant NDUFAF6 cell line was also checked by an immunofluorescence assay. NDUFAF6 protein signal was low compared to controls associated with mitochondrial network disruption. CoC3 treatment partially corrected both NDUFAF6 protein signal and the normal mitochondrial network morphology (Figure 3A). In addition, MitoTracker signal was markedly low in mutant cells compared to controls, suggesting mitochondrial depolarization. As expected, CoC3 treatment was also able to partially increase mitochondrial membrane potential (Figures 3B,C).

### Pterostilbene and Mitochondrial Cofactors Supplementation Improves Cell Bioenergetics in Mitochondrial Mutant Cells

**FIGURE 4:**
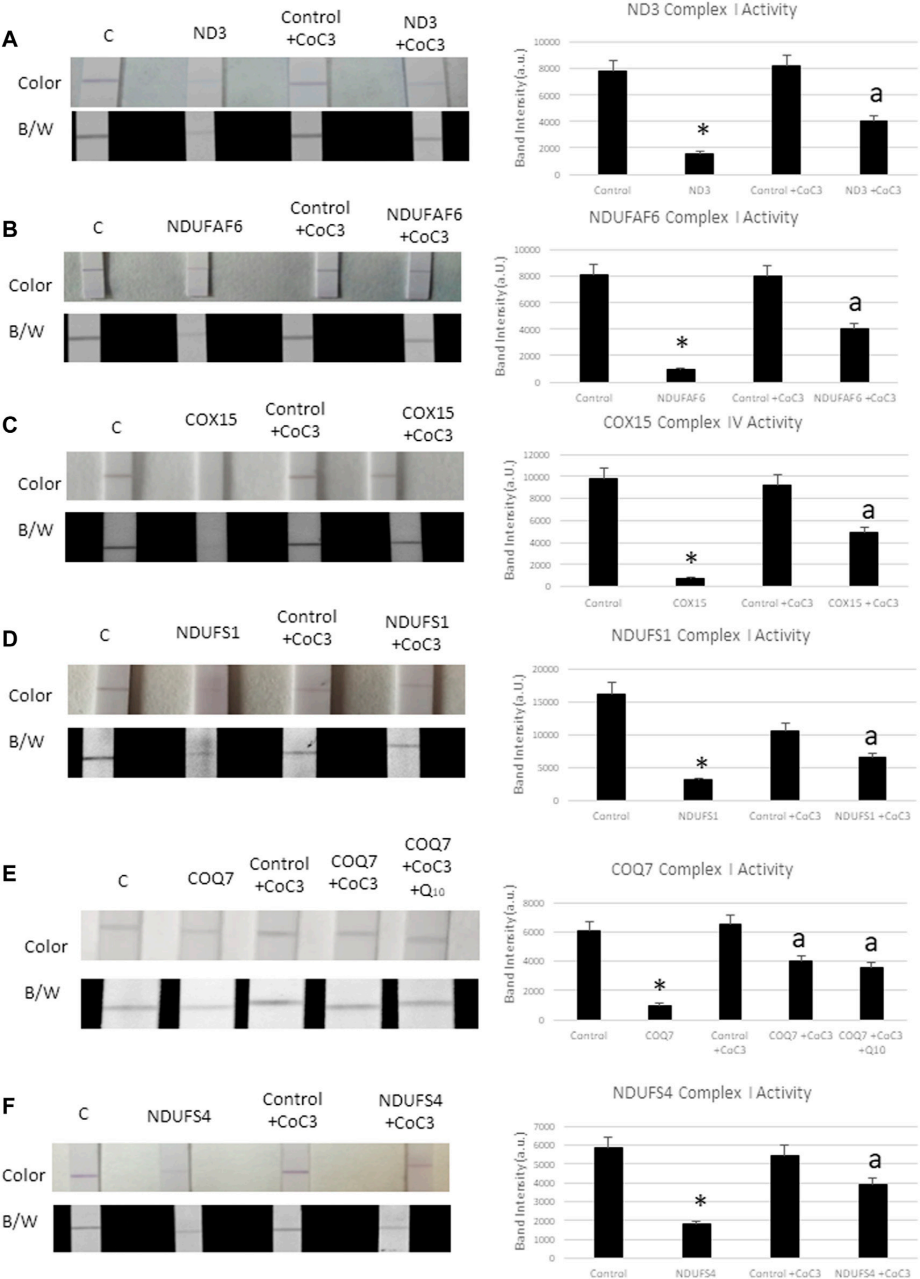
Effect of CoC3 on complex I and complex IV activities in control and mitochondrial mutant fibroblasts. Complex I proteins’ activities were measured using Complex I Enzyme Activity Dipstick Assay Kit from Abcam, with the exception of mutant COX15. In this particular mutation complex IV was measured. Mitochondrial complex activity in mtND3 cells **(A)**, NDUFAF6 cells **(B)**, COX15 cells **(C)**, NDUFS1 cells **(D)**, COQ7 cells **(E)**, NDUFS4 cells **(F)**. Results are shown in the dipstick images both in colour (bluish for complex I and yellowish for complex IV) and black/white. Band intensity was quantified using the Image Lab software. “C” stands for healthy control cells. Fibroblasts were treated for 7 days with CoC3 treatment: 1 μM Pterostilbene, 5 μM nicotinamide, 1 μM riboflavin, 1 μM thiamine, 1 μM biotin, 5 μM lipoic acid and 1 μM l-carnitine. * = *p* < 0.01 between Control and mutant fibroblasts. ^a^ *p* < 0.01 between untreated and treated mutant fibroblasts. a.u. (Arbitrary unit).

To test the efficacy of the CoC3 treatment in improving mitochondrial activity, complex I and complex IV enzyme activity were determined in control and mutant cells. Complex I activity was tested in mtND3 (Figure 4A), NDUFAF6 (Figure 4B), NDUFS1 (Figure 4D), COQ7 (Figure 4E) and NDUFS4 (Figure 4F) cells. The activity of complex IV was tested in mutant COX15 cells (Figure 4C). The activity of both mitochondrial complexes markedly increased after treatment with CoC3 in all mutant cell lines.

As mutant COQ7 cells showed coenzyme Q_10_ deficiency (Supplementary Figure S6), cells were also treated with CoC3 as well as with CoC3 plus 4 μM coenzyme Q_10_ (Figure 4E). However, coenzyme Q_10_ supplementation did not significantly improve the positive effects of CoC3 alone in complex I activity.

**FIGURE 5:**
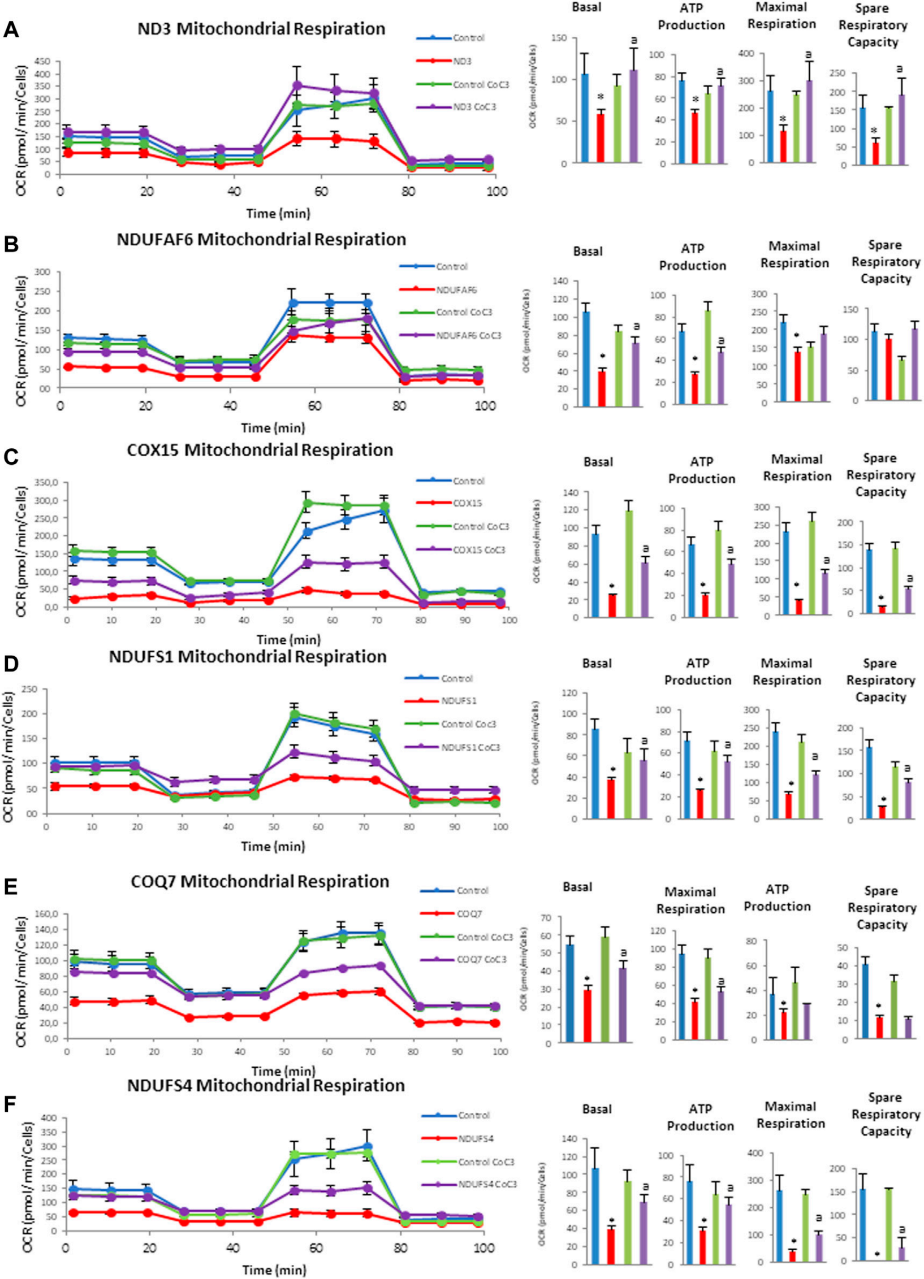
Effect of CoC3 on mitostress bioenergetic assays in control and mitochondrial mutant cell lines. Mitochondrial respiration profile was measured with a Seahorse XFe24 analyzer. Fibroblasts were treated for 7 days with CoC3 treatment: 1 μM Pterostilbene, 5 μM nicotinamide, 1 μM riboflavin, 1 μM thiamine, 1 μM biotin, 5 μM lipoic acid and 1 μM l-carnitine. Mutations in figure panels: mtND3 **(A)**, NDUFAF6 **(B)**, COX15 **(C)**, NDUFS1 **(D)**, COQ7 **(E)**, NDUFS4 **(F)**. * = *p* < 0.01 between Control and mutant fibroblasts. ^a^ *p* < 0.01 between untreated and treated mutant fibroblasts. OCR, oxygen consumption rate.

Next, mitochondrial bioenergetics was assessed using the Mito-stress test assay in an XF24 extracellular flux analyzer (Seahorse Bioscience, Billerica, MA). Similarly, to other pathological alterations, mitochondrial bioenergetics was affected in mutant cell lines in different degrees but they all present a general decrease in mitochondrial activity. Confirming the positive effect previously observed, the supplementation with CoC3 significantly restored mitochondrial bioenergetics in all mutant cell lines (Figures 5A,F).

### UPR^mt^ is Activated by Pterostilbene and Mitochondrial Cofactors Treatment

**FIGURE 6:**
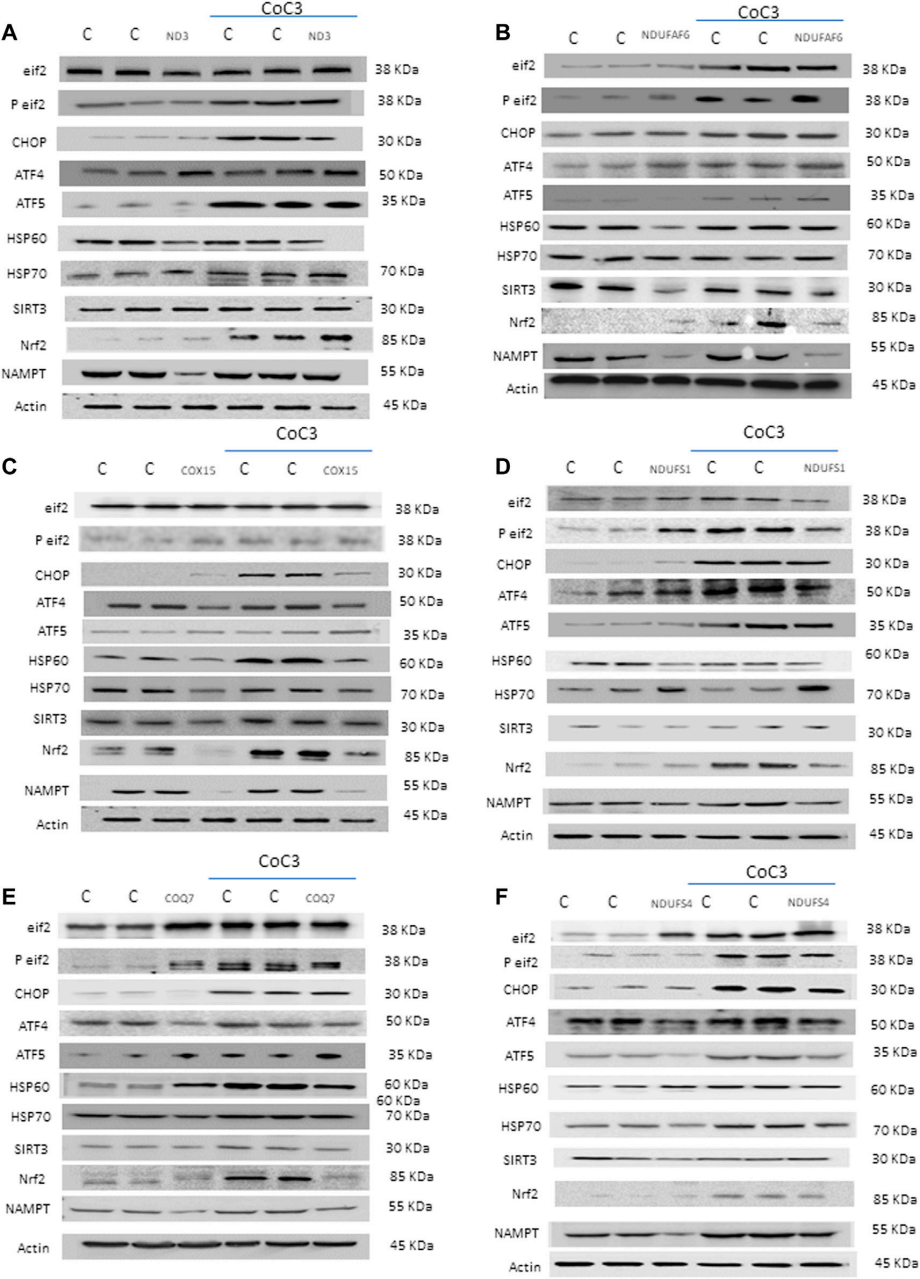
Effect of CoC3 on the expression levels of UPR^mt^-related proteins in control and mitochondrial mutant cell lines. Western blot analysis of several UPR^mt^ -related proteins: Eif2α and P-eif2α proteins as Integrated Stress Response (ISR) markers; ATF4, ATF5 and CHOP proteins as canonical UPR^mt^ proteins; HSP60 and mtHSP70 proteins as chaperones; SIRT3 protein as main target of pterostilbene; Nrf2 protein as a modulator of mitochondrial antioxidant pathways; NAMPT enzyme as NAD^+^/NADH cell metabolism regulator. “C” stands for healthy control cells. Fibroblasts were treated for 7 days with CoC3 treatment: 1 μM Pterostilbene, 5 μM nicotinamide, 1 μM riboflavin, 1 μM thiamine, 1 μM biotin, 5 μM lipoic acid and 1 μM L-carnitine. Mutations in figure panels: mtND3 **(A)**, NDUFAF6 **(B)**, COX15 **(C)**, NDUFS1 **(D)**, COQ7 **(E)**, NDUFS4 **(F)**. A representative actin band is shown for all assays, although loading control was checked for every Western blot. Band densitometry is shown in Supplementary Figure S7.

It is known that the mechanism of action of pterostilbene analogues, such as resveratrol (Houtkooper et al., 2013; Regitz et al., 2016) or piceatannol (Papandreou et al., 2015; Hosoda et al., 2021), is explained by their ability to regulate mitochondrial proteostasis. Thereafter, we decided to explore the possibility of UPR^mt^ activation by pterostilbene plus mitochondrial cofactors (Figure 6). For this, protein expression levels of UPR-related proteins was assessed in treated and untreated cell lines: eif2α and its active form p-eif2α as initiators of the Integrated Stress Response (ISR) (Pakos-Zebrucka et al., 2016; He et al., 2020), Nrf2 as mitochondrial function regulator upon stress conditions (He et al., 2020), CHOP, ATF4 and ATF5 as main UPR^mt^ effectors in humans (Melber and Haynes, 2018; Wang YT. et al., 2019; Jiang et al., 2020), and HSP60 and HSP70 as mitochondrial chaperones (Ruan et al., 2020). Additionally, we examined the expression levels of SIRT3 as a pterostilbene target (Bai et al., 2018; Wang T. et al., 2019) and NAMPT which catalyzes the rate-limiting step in NAD biosynthesis. Application of the CoC3 treatment led to a significant increase in protein expression levels of ISR, UPR^mt^-related proteins and NAMPT in both mutant and control cells (Figures 6A,F; Supplementary Figures S7A–F).

### Pterostilbene and Mitochondrial Cofactors Treatment Improves NAD^+^/NADH Ratio and SIRT3 Activity in Mitochondrial Mutant Cells

SIRT3 is a canonical mitochondrial sirtuin (Zhang J. et al., 2020) able to regulate several mitochondrial proteins (Yang et al., 2016). Aiming to assess the activity of such sirtuin in a mitochondrial disease context we measured NADH levels and the NAD^+^/NADH ratio, given that these are sirtuins cofactors related with cellular fitness (Supplementary Figures S8A–D). We selected mutant mtND3 and NDUFAF6 cells as representatives cell lines and they were treated with CoC3. Our results showed a marked increase of NAD^+^ content and NAD^+^/NADH ratio after CoC3 treatment in both control and mutant cell lines (Supplementary Figures 8C,D) which is associated with increased NAMPT protein expression levels (Figure 6).

**FIGURE 7:**
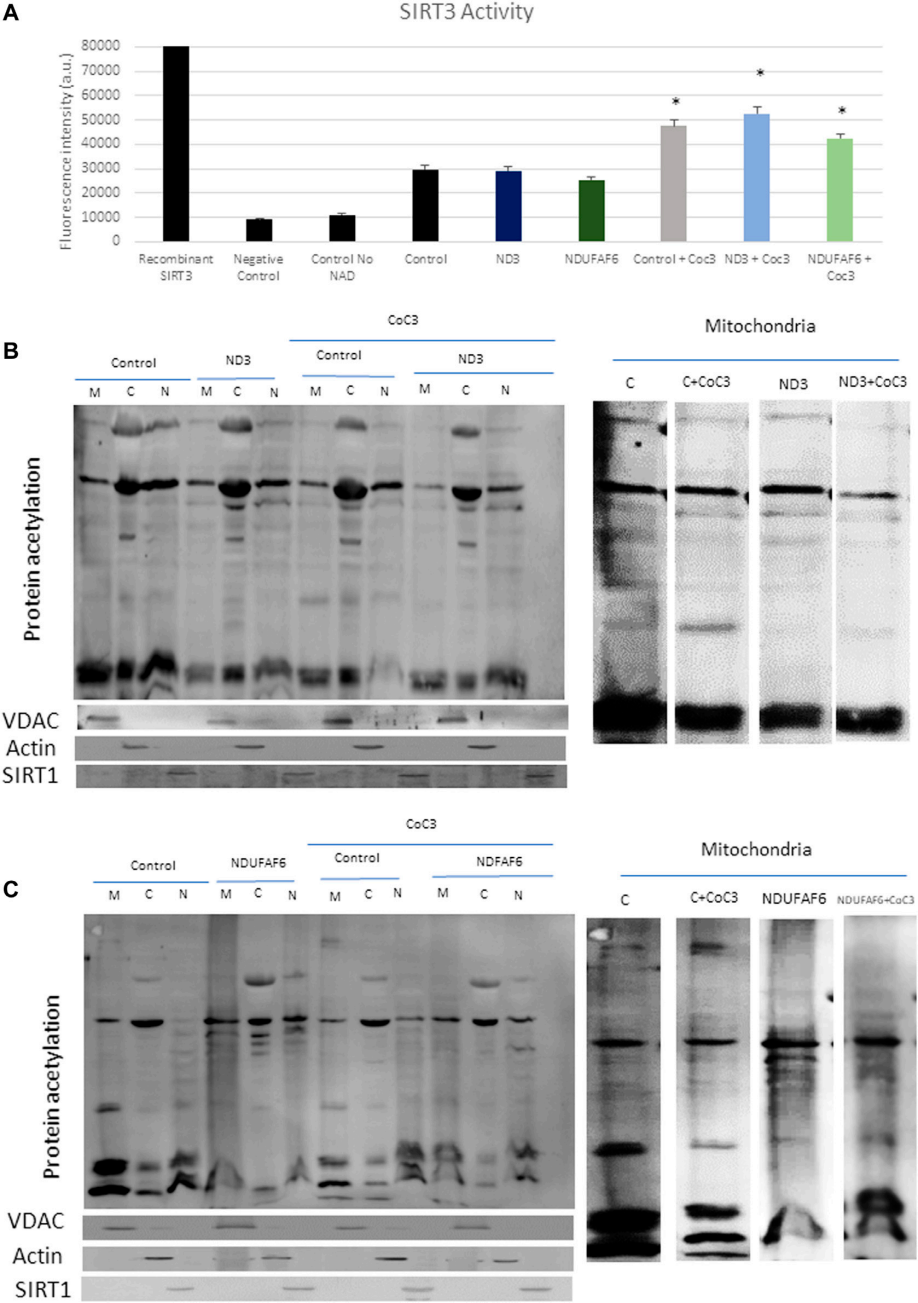
Effect of CoC3 on SIRT3 activity in control and mutant cell lines. SIRT3 activity was determined in mutant mtND3 and NDUFAF6 cells. Total SIRT3 activity was evaluated using the SIRT3 Activity Assay Kit (Fluorometric) from Abcam **(A)**. Pure SIRT3 protein was used as positive control, and no-enzyme and no-NAD (SIRT3 cofactor) samples as negative controls. Fluorescence was measured using a POLARstar Omega plate reader. Western blot of total protein acetylation in mutant mtND3 **(B)** and mutant NDUFAF6 **(C)** fibroblasts. Protein acetylation was assessed in mitochondria, cytoplasm and nuclei fractions. VDAC was used as mitochondrial protein marker; Actin was used as cytoplasm protein marker; SIRT1 was used as nuclear protein marker. Band densitometry is shown in Supplementary Figure S9. Fibroblasts were treated for 7 days with CoC3 treatment: 1 μM Pterostilbene, 5 μM nicotinamide, 1 μM riboflavin, 1 μM thiamine, 1 μM biotin, 5 μM lipoic acid and 1 μM L-carnitine. * = *p* < 0.01 between untreated and treated fibroblasts.

Furthermore, the deacetylase activity of SIRT3 in such patients was measured with two different assays. The first approach consisted in measuring SIRT3 activity through the addition of an acetylated peptide that acts as a fluorescent reporter once deacetylated. Accordingly, SIRT3 activity increased in treated controls and mutant cells (Figure 7A). Second, cells were fractionated into mitochondria, cytoplasm and nucleus and measured acetylation levels in each compartment via Western blot (Figures 7B,C; Supplementary Figures S9A,B). Both in control and mutant cells a significant reduction in mitochondrial proteins’ acetylation was observed upon CoC3 treatment, being such decrease less noticeable for nuclear proteins.

### Pterostilbene and Mitochondrial Cofactors Supplementation has Positive Effects in INs

The fibroblast model provided useful information on the pathophysiology of this disease, however, the most affected cell types in the majority of mitochondrial pathologies are muscle cells and/or neurons (Gorman et al., 2016). Therefore, direct reprogramming of mitochondrial diseases patient-derived fibroblasts into induced neurons (iNs) is a valuable tool to understand the pathogenesis of these disorders. For this reason, control and mutant NDUFAF6 fibroblasts were direct-reprogrammed to iNs. Reprogrammed cells presented a typical neuron-like morphology and positive immunoreactivity against Tau, a microtubule-associated protein primarily found in neuronal axons of vertebrates’ brain. In contrast, unprogrammed cells did not show Tau staining.

Tau+ cells were used to calculate neuronal conversion efficiency (Tau+ cells over the total number of fibroblasts seeded for conversion), which was around 10% in control (9 ± 1.8%) and 9% in mutant NDUFAF6 cells (8 ± 1.9%) cells. Neuronal purity (Tau+ cells over the total cells in the plate after reprogramming) was around 13% (10 ± 2.6%) in control cells and up to 12% (10 ± 2.1%) in mutant NDUFAF6 cells.

The efficacy of CoC3 treatment in mutant NDUFAF6 iNs was then evaluated. NDUFAF6 protein levels were examined by immunofluorescence microscopy (Supplementary Figure S10A). Additionally, mitochondrial network integrity and potential was assessed by MitoTracker Deep Red FM staining. In mutant NDUFAF6 iNs, NDUFAF6 protein and MitoTracker signals were markedly low compared to controls (Supplementary Figures S10B,C). Interestingly, CoC3 treatment partially reverted the disease phenotype by increasing both NDUFAF6 protein and MitoTracker signals on mutant NDUFAF6 iNs as previously seen in fibroblasts.

## Discussion

There is a critical need to find effective treatments for mitochondrial diseases (Mok et al., 2020). Thanks to the advances in next generation sequencing and the rising affordability of this resource, the number of patients diagnosed with these diseases has sharply increased. This has encouraged researchers to focus on therapeutic options for mitochondrial diseases. Some strategies propose the activation of AMPK (Madhavi et al., 2019) or the induction of selective pressure over defective mitochondria (Villanueva Paz et al., 2016). We propose that UPR^mt^ activation and the subsequent improvement in mitochondrial proteostasis and antioxidant activity (Zhu et al., 2021), is a potential alternative therapeutic approach for mitochondrial diseases.

Aiming to find therapeutic candidates we developed a screening culture medium at which cells bearing mitochondrial mutations could not survive unless being treated with the right compounds. Our screenings identified resveratrol and its derivatives as promising treatments, whose efficacy was further boosted by the addition of nicotinamide and other common mitochondrial activity enhancers such as thiamine, riboflavin, L-carnitine and lipoic acid. Amidst resveratrol’s derivatives we chose pterostilbene due to its higher bioavailability, which would make it more suitable for clinical application (Wang and Sang, 2018). Pterostilbene, like resveratrol, is an activator of sirtuins such as SIRT1 as widely reviewed in the literature (Li YR. et al., 2018; Song et al., 2019; Zhu et al., 2020). On the other hand, the activation of SIRT3 by resveratrol has been recently linked with an increase in lifespan (McDonnell et al., 2015) and an improvement in diverse pathologies such as atherosclerosis (Sosnowska et al., 2017), cardiac alterations (Chen et al., 2015; Wang HN. et al., 2020) and cytotoxicity at different cell types (Wang L. et al., 2020; Zhang Q. et al., 2020). Our results confirm pterostilbene’s efficacy potential and demonstrate its ability to activate SIRT3 as well as its mitochondrial deacetylase function. We hypothesize that pterostilbene, through SIRT3 activation, in combination with mitochondrial cofactors could boost antioxidant mechanisms, enhance fatty acids’ β-oxidation, regulate mitochondrial protein quality control and adapt the OXPHOS system, as previously reported in the literature (van de Ven et al., 2017; Silaghi et al., 2021). In general, pterostilbene improves mitochondrial homeostasis and thus palliates the negative impact of mitochondrial mutations.

Although UPR^mt^ activation pathways are not entirely understood, several studies (Weng et al., 2020) have correlated SIRT3 activation with UPR^mt^, and suggest it might be a key factor explaining its ability to increase lifespan (Rose et al., 2017). Indeed, the link between sirtuins and UPR^mt^ activation was recently confirmed in animal models (Jovaisaite and Auwerx, 2015; Mendelsohn and Larrick, 2017). These studies additionally suggested their involvement in the maintenance of mitochondrial proteostasis. UPR^mt^ not only controls the degradation of aberrant proteins but also balances protein import and export in the mitochondrial compartment and enhances protein folding capacity (Ji et al., 2020). These mechanisms improve mitochondrial function and overall cellular stress adaptation (Mora et al., 2017). In this study we could demonstrate that pterostilbene activates UPR^mt^ in mitochondrial mutant fibroblasts leading to a significant improvement of cellular bioenergetics. We believe that by activating UPR^mt^ pterostilbene boosts chaperones’ activity, which would then stabilize the mutant protein. In this scenario, such protein would not be immediately degraded and would remain in the cell with a residual function. The slight increase in functionality of the aforementioned protein would be sufficient to enable cell survival in the restrictive screening medium. In line with this, SIRT3 ability to enhance folding and stability of mitochondrial proteins has already been thoroughly described (Gibellini et al., 2014; Lu et al., 2015).

Another key point to consider is the fact that pterostilbene plus mitochondrial cofactors supplementation improved the NAD^+^/NADH ratio and NAMPT expression levels. NAD^+^ is a vital cofactor for sirtuins’ function (Imai and Guarente, 2014). Throughout ageing NAD^+^ decrease and so does sirtuin activity (Ramsey et al., 2008; Chang and Guarente, 2013). This phenomenon leads to progressive impairment of mitochondrial function (Gomes et al., 2013) that eventually results in mitochondrial dysfunction similar to that present in mitochondrial (Schondorf et al., 2018) or neurodegenerative diseases (Lautrup et al., 2019). Previous studies confirm that supplementation with NAD^+^ precursors such as nicotinamide restores NAD^+^ levels and improves cell bioenergetics and function (Belenky et al., 2007; Yoshino et al., 2011; Canto et al., 2012). The relevance of SIRT3 as compensatory mechanism can be confirmed considering that its inhibition by 3-TYP supress mutant cells survival in galactose medium even with CoC3 treatment (Supplementary Figure S3).

In summary, pterostilbene in combination with mitochondrial cofactors treatment activates SIRT3 and UPR^mt^ as compensatory mechanisms as well as enhance sirtuins’ levels and mitochondrial activity in several cell models of mitochondrial diseases. Recent findings have led to an increase in the relevance of UPR^mt^ activation as a novel therapeutic approach for the treatment of mitochondrial diseases and related conditions (Poveda-Huertes et al., 2021) (Ji et al., 2020). Interestingly, this potent compensatory pathway may not be active in cells bearing a range of different mutations that secondarily affect mitochondrial proteostasis (Pitera et al., 2019). This study provides compelling evidence to suggest that the activation of UPR^mt^ with drugs such as pterostilbene in combination with mitochondrial cofactors promote the improvement of mitochondrial function in fibroblasts with several mitochondrial mutations. Moreover, our findings corroborate that pterostilbene activates sirtuins and that, by doing so, it can boost the activation of UPR^mt^, as proposed by other authors (Weng et al., 2020). The application of personalised screening with UPR^mt^ activators opens a new window of possibilities for the treatment of genetic mitochondrial pathologies.

## Data Availability

The original contributions presented in the study are included in the article/Supplementary Material, further inquiries can be directed to the corresponding author.

## Glossary

AMPK: AMP-activated protein kinase
ATF4: Activating Transcription Factor 4
ATF5: Activating Transcription Factor 5
BSA: Bovine Serum Albumin
CHOP: C/EBP homologous protein
COX15: Cytochrome C oxidase Assembly Homolog 15
COX4: Cytochrome C oxidase subunit four
DMEM: Dulbecco’s Modified Eagle Medium
DMQ: (6-demethoxyubiquinone)
Eif2α: Eukaryotic Initiation Factor 2 alpha
FBS: Fetal Bovine Serum
FCCP: Carbonyl cyanide-p-trifluoromethoxyphenylhydrazone
FDA: Food and Drugs Administration
HEPES: 4-(2-hydroxyethyl)-1-piperazineethanesulfonic acid
HSP60: Heat Shock Protein 60
iNs: Induced Neurons
ISR: Induced Stress Response
mtCO2: Mitochondrial Cytochrome C oxidase subunit II
mtDNA: Mitochondrial DNA
mtHSP70: Mitochondrial encoded Heat Shock Protein 70
mtND1: Mitochondrial encoded NADH-ubiquinone oxidoreductase chain subunit one
mtND3: Mitochondrial encoded NADH-ubiquinone oxidoreductase chain subunit three
NADH: Nicotinamide adenine dinucleotide
NAMPT: Nicotinamide phosphoribosyltransferase
NDUFA9: NADHUbiquinone oxidoreductase Subunit A9
NDUFAF6: NADHubiquinone oxidoreductase complex assembly factor 6
NDUFS4: NADHUbiquinone oxidoreductase Subunit S4
Nrf2: Nuclear Respiratory Factor 2
P- Eif2α: Phosphorilated Eukaryotic Initiation Factor 2 alpha
PBS: Phosphate Buffer Saline
ROS: Reactive Oxygen Species
SIRT1: Sirtuin one
SIRT3: Sirtuin 3
3-TYP: (3-(1H-1,2,3-triazol-4-yl) pyridine)
UPRmt: Mitochondrial Unfolded Protein Response
VDAC: Voltage Dependant Anion Channel
