# Supplementary material for: Pterostilbene in Combination With Mitochondrial Cofactors Improve Mitochondrial Function in Cellular Models of Mitochondrial Diseases
Source: Front Pharmacol. 2022 Mar 18;13:862085. doi: 10.3389/fphar.2022.862085 (PMC8971666; doi:10.3389/fphar.2022.862085)
Supplement: Supplementary file 1 [file DataSheet1.docx]

*
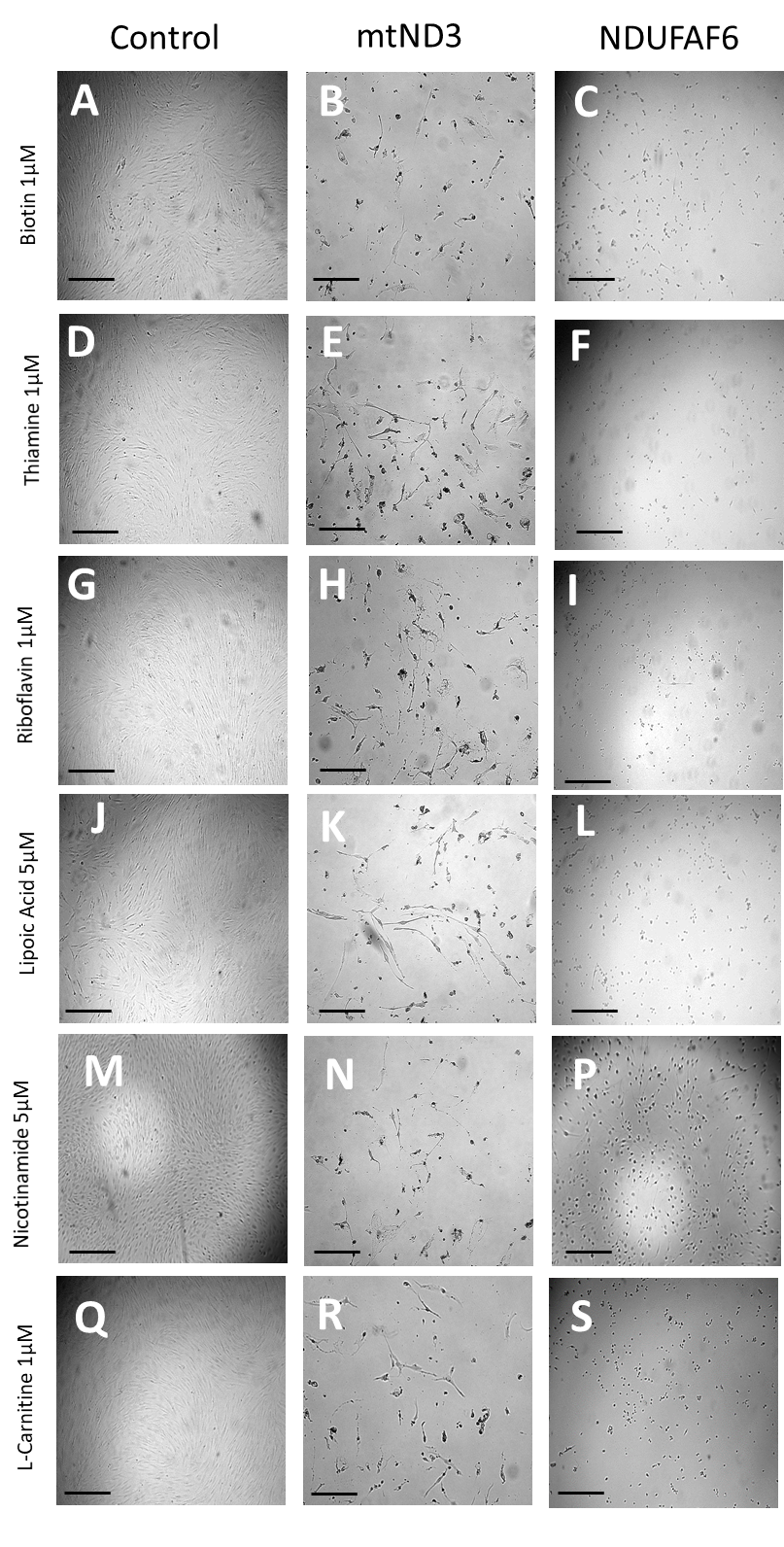
*

**Supplementary Figure 1.** Effect of single compounds of CoC3 on cell survival in stress medium. Cells were initially seeded in DMEM high glucose. After 3 days, glucose medium was changed to galactose and treatments were applied. Images were acquired right after changing the medium and 72h later. Control cells showed no differences in proliferation rate **(A, D, G, J, M, Q)**. However, none of the treatments allowed the survival of mutant ND3 cells (**B, E, H, K, N, R**) nor mutant NDUFAF6 cells (**C, I, F, L, P, S**). Quantification of cellular proliferation is shown in Supplementary Figure 2. Scale bar=40 μm.


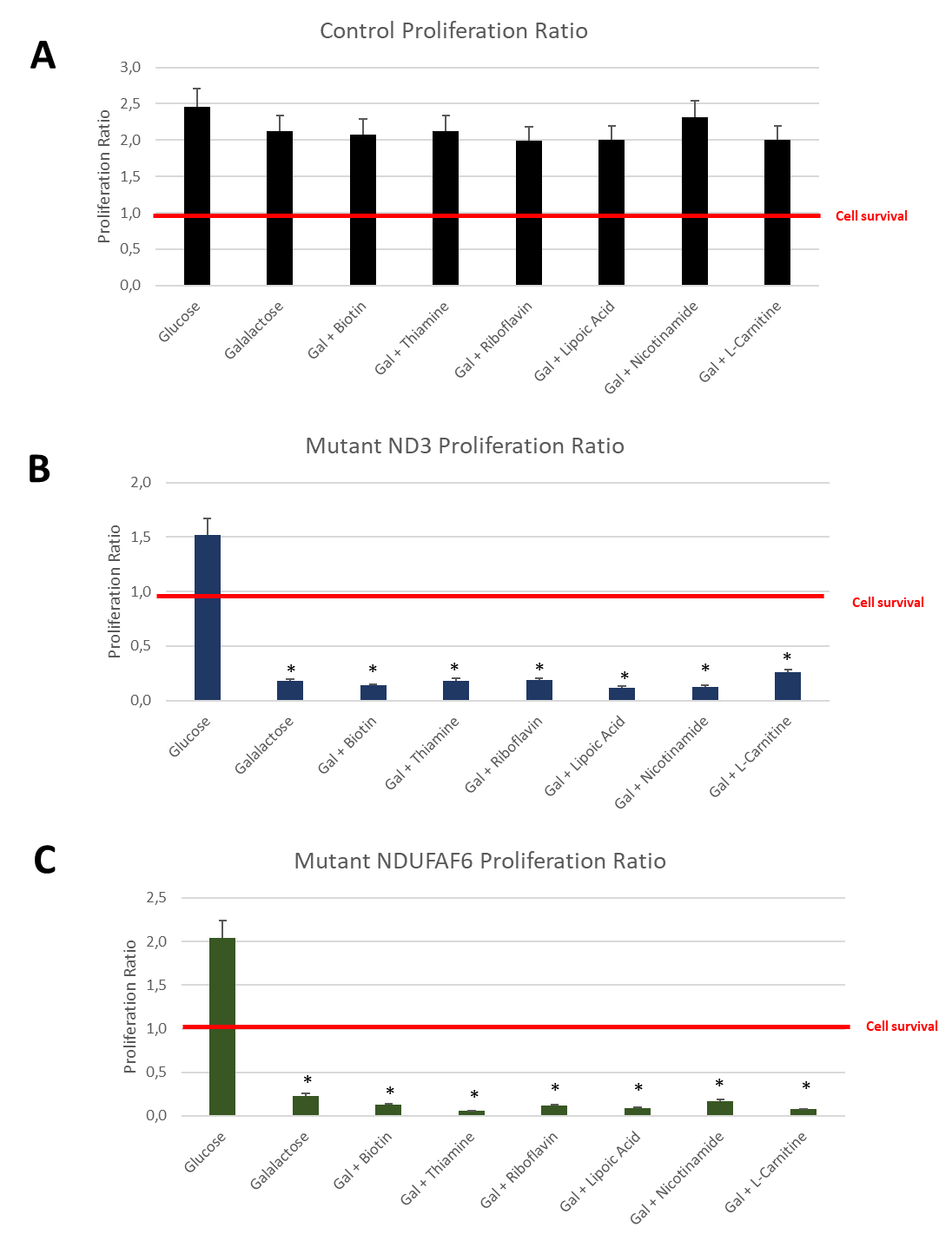


**Supplementary Figure 2.** Quantification of cellular proliferation in galactose medium of control and mitochondrial mutant fibroblasts treated with single compounds of CoC3. Cell proliferation rate was obtained from the quotient: Nº Cells at T72h/ Nº cells at T0h. Results close to 1 imply cell survival, below 1 indicate cell death and higher than 1 cell proliferation. Control cells **(A)**; Mutant mtND3 cells **(B)**; Mutant NDUFAF6 cells **(C)**. The data represents the mean± SD of 3 independent experiments. *p<0.01 between glucose and galactose medium; ^a^p<0.01 between galactose medium and treatments.


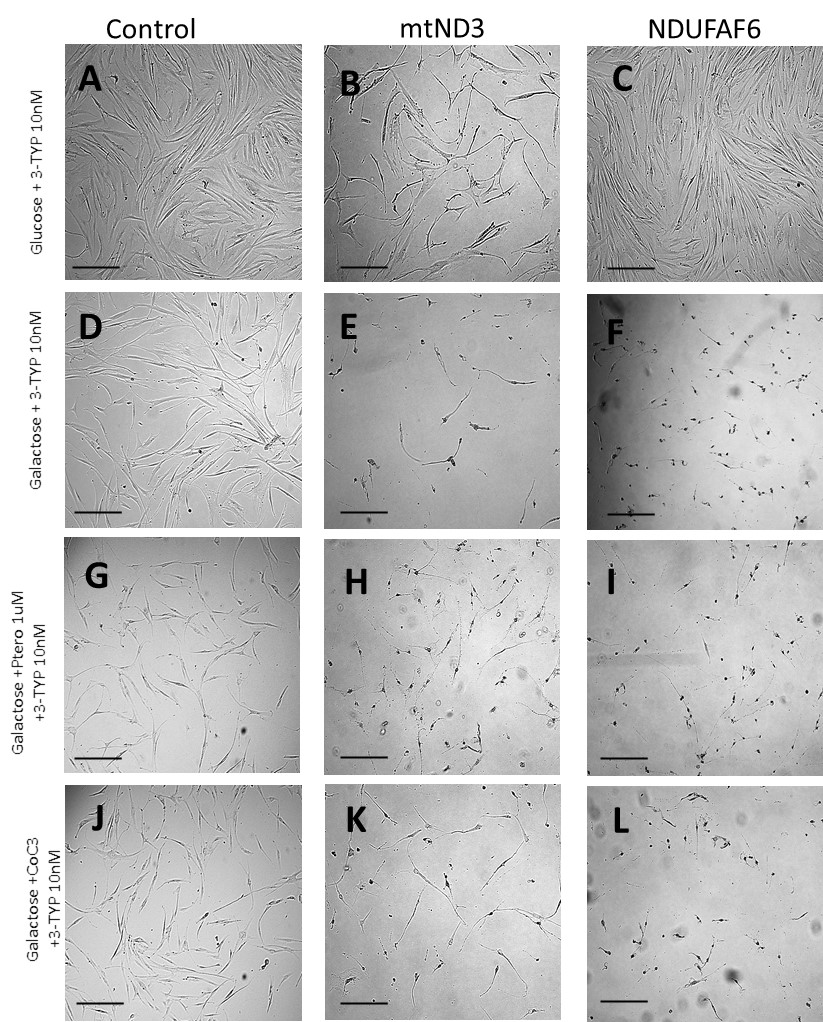


**Supplementary Figure 3.** Effect of SIRT3 inhibition by 3-TYP on cell survival in galactose medium. Cells were initially seeded in DMEM high glucose. After 3 days, glucose medium was changed to galactose and 10nM 3-TYP treatment was applied. Images were acquired right after changing the medium and 72h later. Controls and mutant NDUFAF6 cells showed no differences in proliferation rate in glucose medium **(A, C)**, although, mutant ND3 cells’ growth rate is noticeably slower **(B)**. Control cells’ growth is partially compromised by the supplementation of galactose medium with 3-TYP **(G, J)**. In presence of 3-TYP, both mutant cell lines underwent cell death after 72h of culture in galactose medium **(E, F, H, I)** even with the supplementation with CoC3 **(K, L)**. CoC3 (1μM Pterostilbene, 5μM nicotinamide, 1 μM riboflavin, 1 μM thiamine, 1 μM biotin, 5 μM lipoic acid and 1 μM L-carnitine). Quantification of cellular proliferation is shown in Supplementary Figure 4. Scale bar=40 μm.


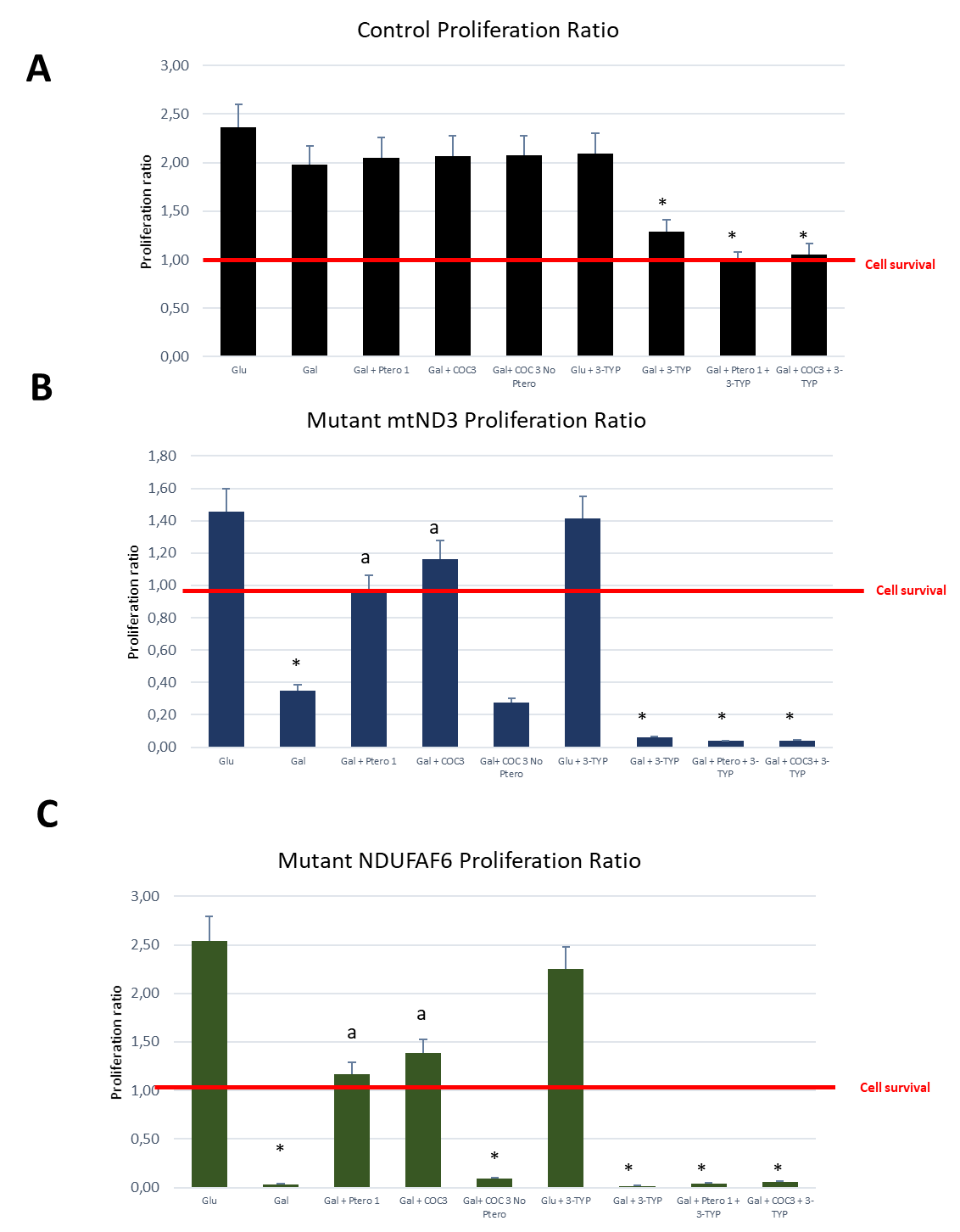


**Supplementary Figure 4.** Quantification of cellular proliferation in glucose and stress medium of control and mitochondrial mutant fibroblasts. Cell proliferation rate was obtained from the quotient: Nº Cells at T72h/ Nº cells at T0h. Results close to 1 imply cell survival, below 1 indicate cell death and higher than 1 cell proliferation. Control cells **(A)**; Mutant mtND3 cells **(B)**; Mutant NDUFAF6 cells **(C)**. The data represents the mean± SD of 3 independent experiments. CoC3 (1μM Pterostilbene, 5μM nicotinamide, 1 μM riboflavin, 1 μM thiamine, 1 μM biotin, 5 μM lipoic acid and 1 μM L-carnitine). TYP-3 (10nM) was used as SIRT3 inhibitor. Glu, Glucose Medium; Gal, Galactose Medium; Ptero, Pterostilbene 1μM. *p<0.01 between glucose and galactose medium; ^a^p<0.01 Galactose medium and treatments.


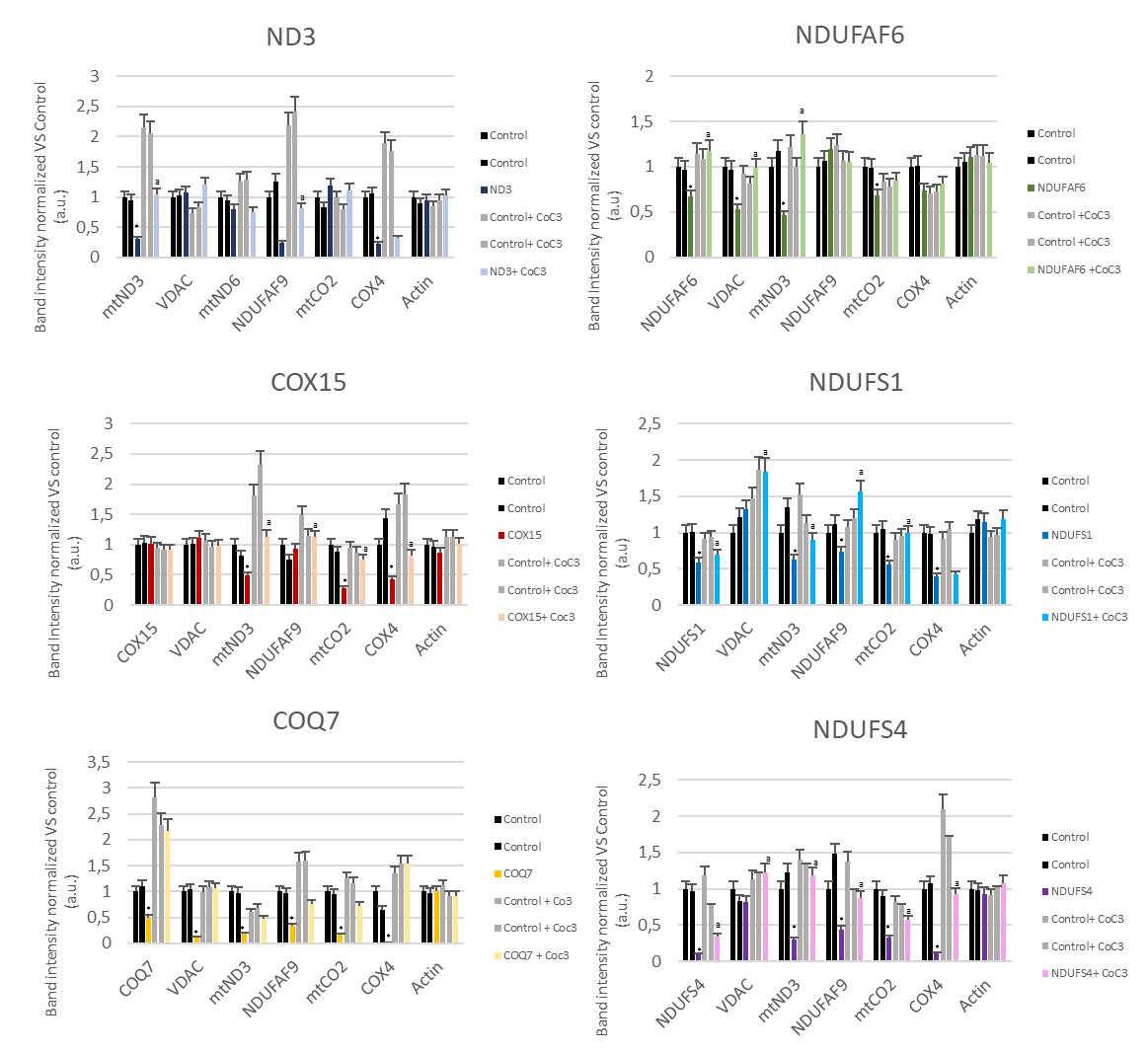


**Supplementary Figure 5.** Western blotting quantification of figure 2. Band densitometry of Western blot shown in Figure 2. Mutations in figure panels: mtND3 **(A)**, NDUFAF6 **(B)**, COX15 **(C)**, NDUFS1 **(D)**, COQ7 **(E)**, NDUFS4 **(F)**. Data were normalized to actin. Bar graphs represent the mean±SD of 3 normalized independent experiments. *p<0.05 between control and mutant cells; ^a^p<0.05 between non-treated and treated conditions. A.U. arbitrary units.


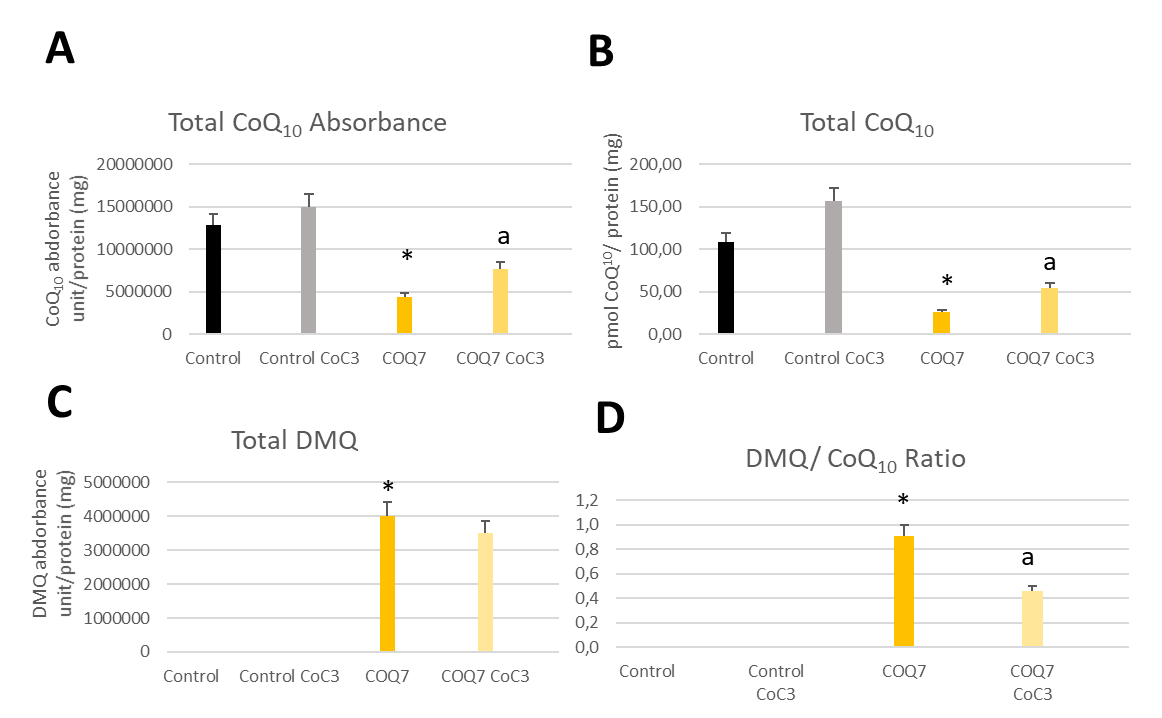


**Supplementary Figure 6.** Coenzyme Q_10_ and DMQ (6‐demethoxyubiquinone) content in mutant COQ7 fibroblasts. Since mutant COQ7 cells are unable to synthesize CoQ_10_ , they accumulate the COQ_10_ precursor DMQ. After CoC3 treatment, mutant COQ7 DMQ levels are decreased and COQ_10_ levels are increased. CoQ_10_ and DMQ content were measured by HPLC using the UV-VIS detection method. Total CoQ_10_ absorbance **(A)**; Total CoQ_10_ content **(B)**; Total DMQ content **(C)**; DMQ/ CoQ_10_ Ratio **(D)**. *p<0.05 between control and mutant COQ7 cells. Data represent the mean±SD of 3 independent experiments. ^a^p<0.05 between non-treated mutant COQ7 and treated mutant COQ7 cells.


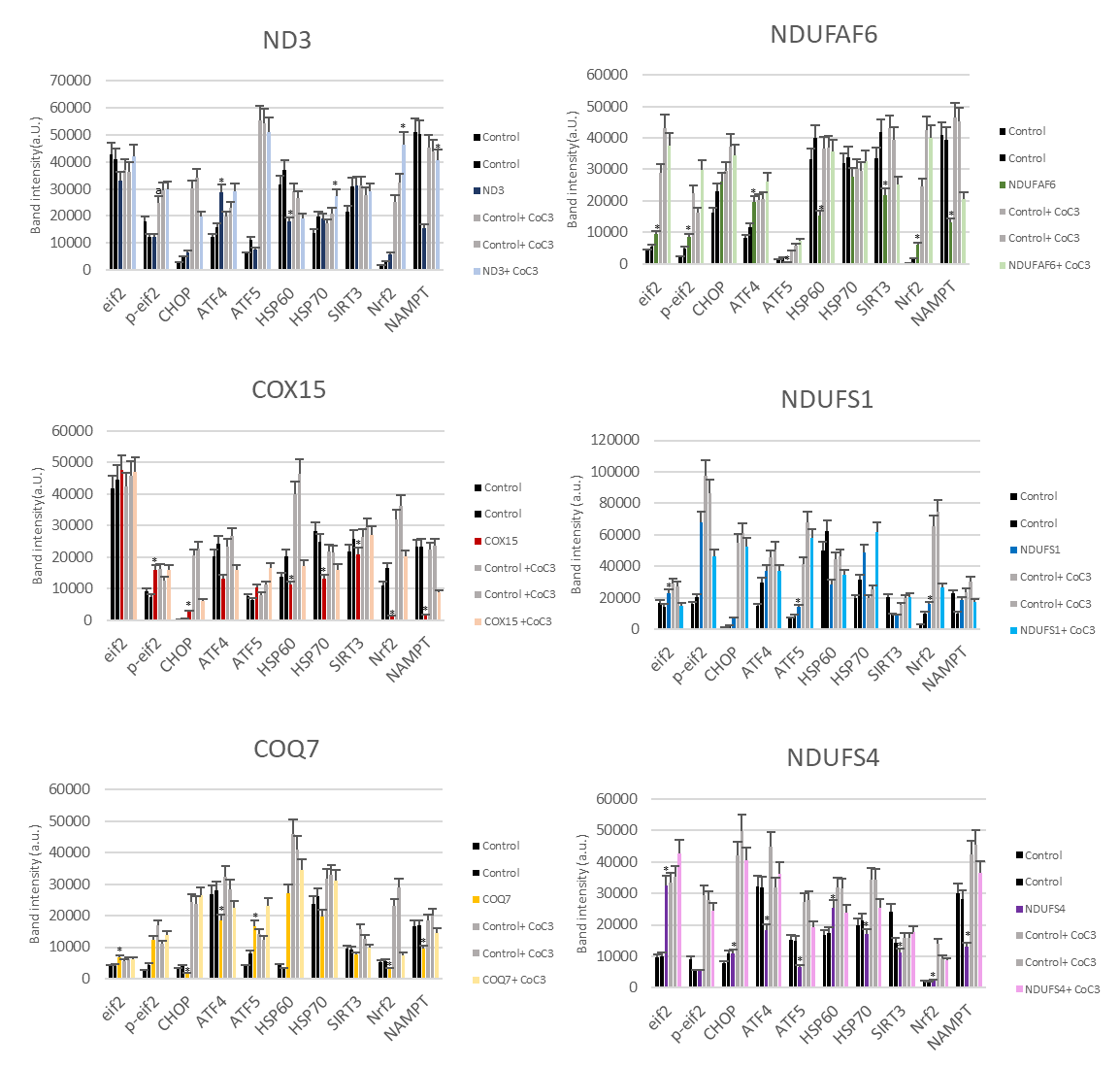


**Supplementary Figure 7.** Western blotting quantification of figure 6. Band densitometry of Western blots shown in Figure 6. Mutations in figure panels: mtND3 **(A)**, NDUFAF6 **(B)**, COX15 **(C)**, NDUFS1 **(D)**, COQ7 **(E)**, NDUFS4 **(F**). Bar graphs represent the mean±SD of 3 independent experiments. *p<0.05 between control and mutant cells; ^a^p<0.05 between non-treated and treated conditions. A.U., arbitrary units.


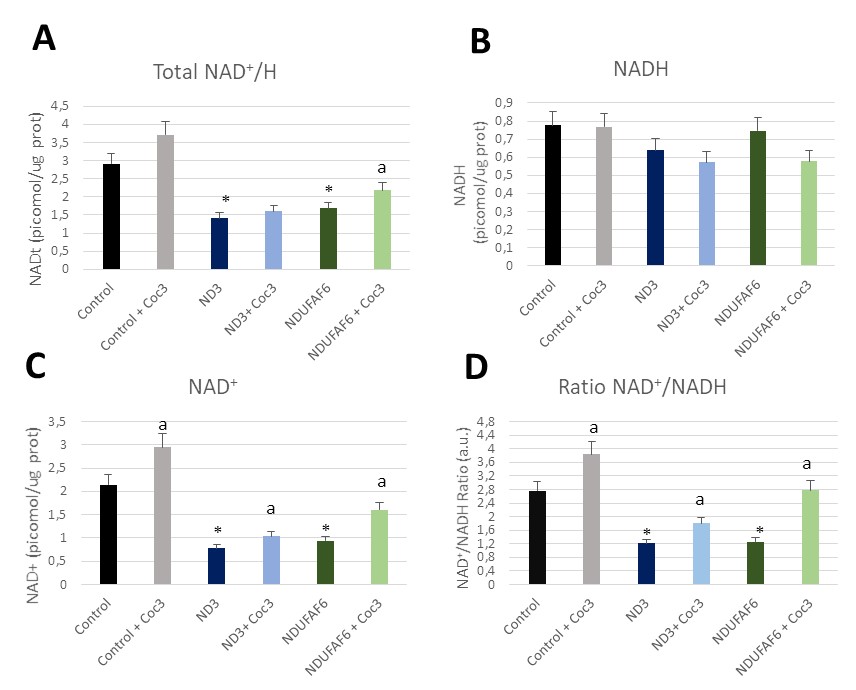


**Supplementary Figure 8.** Effect of CoC3 on cellular NAD^+^/NADH in control and mutant cell lines. The assay was performed in control and mutant ND3 and NDUFAF6 cell lines. NAD^+^/NADH assay was performed using the NAD^+^/NADH Assay Kit from Abcam. NADt (total NAD^+^ and NADH) **(A)** and NADH **(B)** content were quantified via comparison with the standard NADH. NAD^+^ was calculated by subtraction (NADt-NADH) **(C)** and NAD/NADH ratio by the equation (NADt-NADH)/NADH (D). Fibroblasts were treated for 7 days with CoC3 Treatment: CoC3 treatment: 1μM Pterostilbene, 5μM nicotinamide, 1 μM riboflavin, 1 μM thiamine, 1 μM biotin, 5 μM lipoic acid and 1 μM L-carnitine. Results represent the mean±SD of 3 independent experiments. *=p<0.01 between Control and mutant fibroblasts. ^a^p<0.01 between untreated and treated mutant fibroblasts.


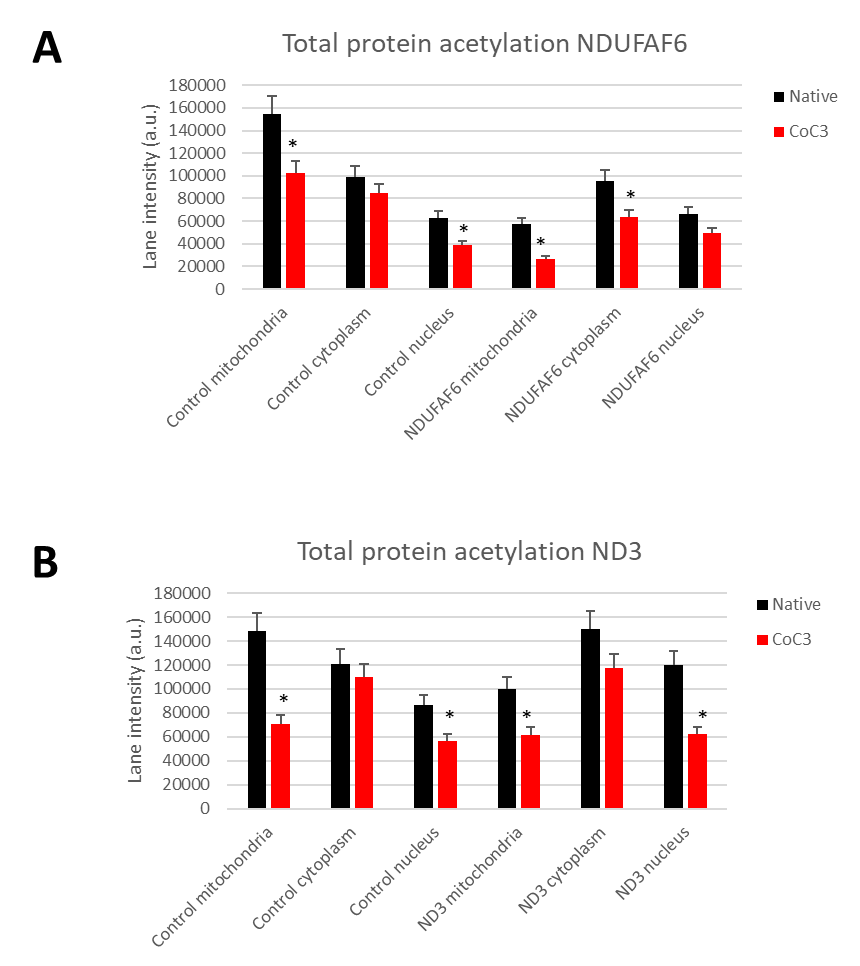


**Supplementary Figure 9.** Western blotting quantification in figures 6B and 6C. Band densitometry of Western blot shown in Figure 6B and 6C. Mutations in figure panels: mtND3 **(A)** and NDUFAF6 **(B)**. Data represent the mean±SD of 3 independent experiments. *p<0.05 between non-treated and treated conditions. A.U., arbitrary units.


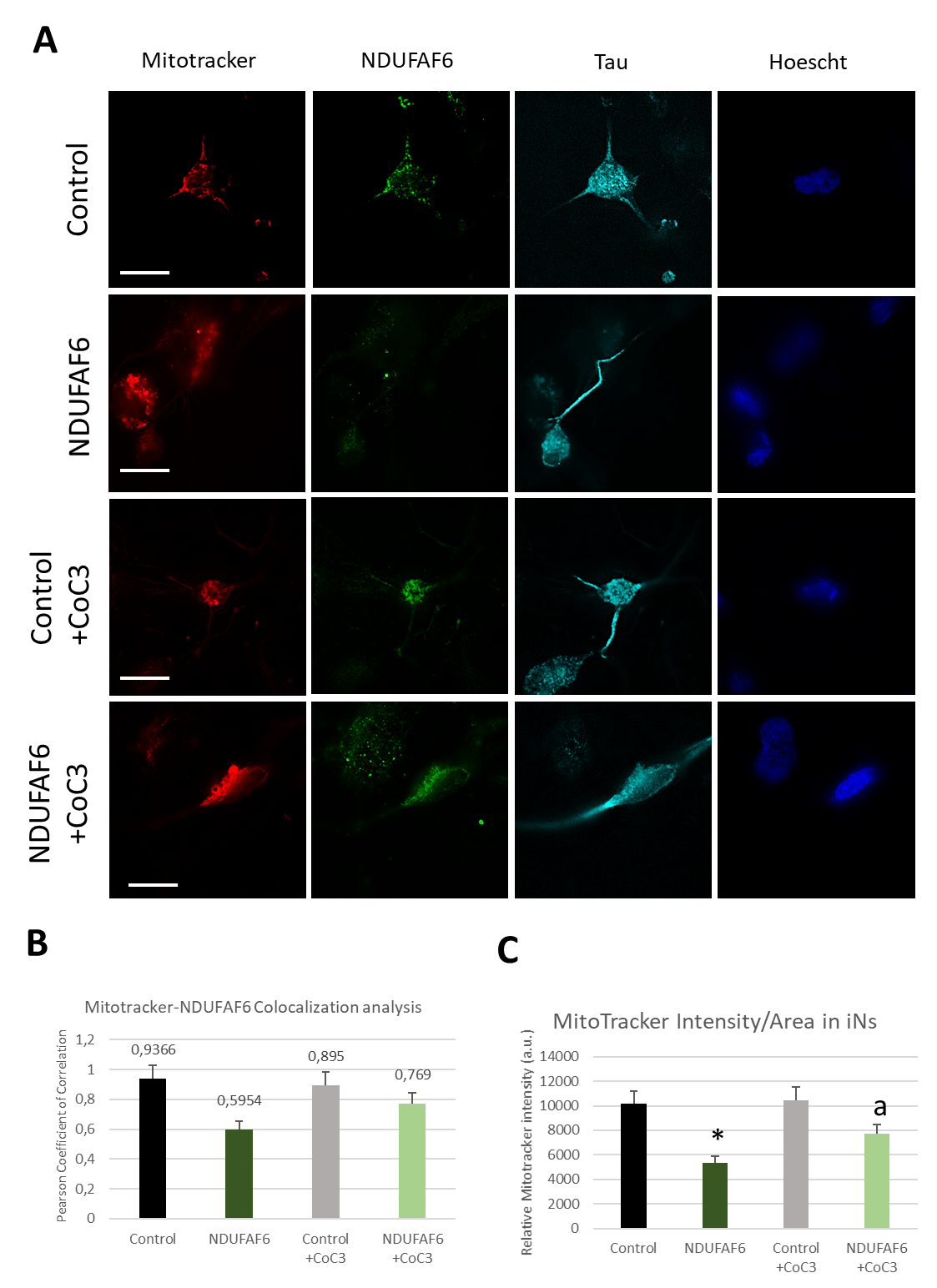


**Figure 10.** Effect of CoC3 on NDUFAF6 protein expression levels, and mitochondrial network morphology in control and mutant NDUFAF6 induced neurons (iNS). Control and mutant NDUFAF6 iNS were incubated with Mitotracker DeepRed FM 100nM for 45 minutes. Then, cells were fixed and immunostained with NDUFAF6 (NADH: Ubiquinone Oxidoreductase Complex Assembly Factor 6) and Tau (neuronal marker) antibodies **(A)**. Subsequently, they were examined by fluorescence microscopy. Nuclei were revealed by Hoescht 1μg/ml staining. Colocalization analyses **(B)** and MitoTracker **(C)** intensity measurements were performed using softWoRx and ImageJ softwares. *p<0.05 between control and mutant NDUFAF6 cells; iNs were treated with CoC3 for 7 days. CoC3 treatment: CoC3 treatment: 1μM Pterostilbene, 5μM nicotinamide, 1 μM riboflavin, 1 μM thiamine, 1 μM biotin, 5 μM lipoic acid and 1 μM L-carnitine. Scale bar=15 μm.
